# Application of Radar Measurements in Neural-Dynamic and Game-Based Safe Ship Controls in Multi-Object Passing Situations

**DOI:** 10.3390/s26196087

**Published:** 2026-09-25

**Authors:** Józef Lisowski

**Affiliations:** Department of Autonomous Systems, Faculty of Computer Science, Gdynia Maritime University, 81-225 Gdynia, Poland; j.lisowski@umg.edu.pl

**Keywords:** sensing in safety navigation development, artificial intelligence, safe control, game control, collision risk

## Abstract

**Highlights:**

**What are the main findings?**

- Synthesis of neural-dynamic and cooperative and non-cooperative game-based control methods for ensuring ship safety using the sensing of collision risk.
- Assessment of the sensitivity of safe ship controls to changes in visibility conditions at sea.

**What are the implications of the main findings?**

- Adaptation of an appropriate safe control method to the complexity of the navigational situation.
- Improvement of maritime navigation safety.

**Abstract:**

Radar sensors, which enable the identification of the navigational situation and a preliminary assessment of collision risk, form the basis for safe ship control. This paper presents the author’s quantitative methods for assessing collision risk in the context of multi-ship navigation, and then synthesizes safe control methods. The author proposes four neural domain variants and three mathematical models of collision risk. Proprietary methods are presented: neural-dynamic control (NDC) and game-based control (GC). An experimental comparison of these control methods is conducted using data from real-world navigation scenarios. Therefore, under favorable traffic conditions, the NDC method proved to be the most effective, while the GC method facilitated effective cooperative and non-cooperative management in situations of restricted ship traffic. The safe control methods proposed in this study will contribute to increased navigation safety, particularly in situations of high ship traffic density and challenging environmental conditions.

## 1. Introduction

The most important task in ship design and construction is the development of algorithms and computer programs for their safe control [1]. For this purpose, the ship must be equipped with a computer system, measurement sensors, and navigation devices, from which the recorded parameters are processed and used to control the ship. The authors of [2] concluded that existing marine mobility structures lack the necessary sensors to avoid emergency situations, such as floods, oil spills, or health hazards, which require effective monitoring. Sensor energy efficiency, data processing, sensor fusion methodologies, and accurate descriptions of the sea state with environmental monitoring using unmanned vehicles are crucial.

The sensing system proposed in [3] utilizes optical and radar data collected from satellite imagery sensors to investigate the activity patterns of maritime vessels. This system utilizes dedicated processing units to discover targets from images based on the vessel type, classify these vessel classes according to their geometric and dispersion characteristics, evaluate their kinematics, assess their navigation behaviors, and predict their routes. In [4], multiple dimensions of sensing are examined using multispectral and multi-sensor technologies, and oceanic phenomena observed via multispectral and search and rescue (SAR) imagery concerning oceans, sea ice, and ship surveillance are analyzed for tracking and navigation needs. A comparative analysis of algorithms designed for collision avoidance and real-time ship routing using radar sensing to identify ship echoes is presented in [5]. The development of an intelligent navigation system for vessels is grounded in a risk assessment that includes collaboration between ships and shore facilities, as well as the evolving risks linked to smart ship navigation through various sensing methodologies, navigational situations, and event chain implications, as discussed in [6]. Sensing facilitates maritime surveillance by enabling the detection and identification of marine traffic from space, utilizing data sourced from satellites. The validation of vessel detections via space-based imagery can be supported by information gathered from the AIS, as noted in [7].

In summary, the above studies address separate types of sensing: ship motion, collision risk, and danger domains, and the less multi-ship and game nature of the safe steering process. This paper, instead of combining the above sensing tasks, leads to their use in the synthesis of adequate methods for safe ship control.

The aim of this work is to demonstrate that, by measuring the process state using ARPA radar followed by selected artificial intelligence and game theory methods, it is possible to synthesize safe control methods in situations where many ships pass each other in different navigation conditions.

The main achievements of this work include the following:

- Synthesis of neural-dynamic and cooperative and non-cooperative game-based control methods for ensuring ship safety using the sensing of collision risk;
- Assessment of the sensitivity of safe ship controls to changes in visibility conditions at sea.

This paper is structured as follows. Section 2 presents work related to the topic of the paper. Section 3 first presents the task of safe ship control based on information from on-board sensors, and then the methodology of synthesizing the control methods and an analysis of their sensitivity are provided. Section 4 presents the results of the simulation studies of these methods in three real-world navigation situations. Section 5 synthesizes the effectiveness of the new control methods presented in the paper. The conclusions are presented in Section 6.

## 2. Related Work

The concept of the ship domain is crucial in the context of intelligent collision avoidance systems and maritime traffic engineering; publication [8] presents traditional ship domain models while discussing their practical applications.

In [9], a methodology for identifying risks in ship navigation merges the concept of the ship domain with sensing data and AIS inputs to enhance prediction accuracy regarding collision risks in intricate waterways. The model of the ship domain is developed using a density map derived from AIS data. Furthermore, reference [10] presents a collision risk model that utilizes the ship domain alongside relevant parameters associated with collision risks, which analyzes encounters between ships through five variables: degrees of domain infringement, relative speeds between vessels, course combinations, arena violations, and complexity during encounters. Traditional models assessing collision risks based on the distance at closest point of approach (DCPA) or time to closest point of approach (TCPA) fall short in effectively estimating collisions or planning avoidance strategies. Therefore, an elliptical dynamic ship domain was designed in [11] to enable adaptation to varying speed and maneuverability features. Navigating potential collision hazards with vessels requires making judgments in an environment of uncertainty, especially when issues such as collision risks or safe speeds are considered. Using fuzzy logic, a novel artificial intelligence (AI) system was shown to be capable of finding safe routes for ships facing potential collisions in [12]. Ref. [13] details relevant development steps, such as analyzing the collision risk between ships, identifying crucial points in traffic, and determining how to plan a route correctly to avoid collisions. Based on [14], we present proposals for the identification of hazards in conjunction with ML algorithms for adverse weather. For collision risk assessment, documented incidents have been examined along with their environmental impacts, for which common analytical frameworks (e.g., probabilistic methods) have been used (for example, in [15]). The results of the research presented in [16] suggest that collision risk prediction can be performed based on DL models using long-term memory networks and Bayesian assessments. Using the hazard–exposure–sensitivity model, pixel-wise navigational risk maps were presented that visualize high-risk areas, mainly near major ports and their access channels; the highest exposure to navigational hazards is recorded for cargo ships, followed by tankers, tugboats, etc. [17]. In [18], a literature review focused on the identification of navigational threats for intelligent vessels was presented, which covered four domains, namely environmental hazards, management practices, equipment reliability, and the effectiveness of navigation systems. Based on the data and research context of maritime autonomous surface ship (MASS) operations, risk assessment methods relevant to MASS operations use a layered approach that combines failure mode analysis with Dempster–Shafer evidence theory and Bayesian networks, as described in [19]. Reference [20] also presents a model seeking to flexibly prioritize failure modes and improve safety controls through the functionalization of autonomous navigation systems. The authors of another study used an approach that combines aspects of game theory, where ships at risk are presented as individual players who plan strategically and independently in a game context using game rules [21]; when their vessel trajectories change, such decisions are framed as strategic decisions, including optimization and adherence to safety and socio-economic norms, as well as socio-economic constraints influencing ship actions.

Previous studies have overlooked the possibility of accounting for

- The navigator’s subjectivity in assessing the safety level of a navigational situation by employing a neural network that generates dynamic domains for passing vessels—with sizes that vary according to collision risk;
- The degree of cooperation between vessels in avoiding collisions by applying a multi-player matrix game model.

Therefore, the author of this paper adopts the thesis that the application of neural-dynamic and game-based controls in multi-ship passing situations improves navigation safety compared to the commonly used basic safe control utilizing the ARPA anti-collision system.

## 3. Methodology

In multi-vessel situations, utilizing information from onboard sensors in combination with appropriate safety control techniques based on artificial intelligence and game theory enables safe ship control. This approach takes into account previously overlooked factors affecting navigational safety and significantly reduces the likelihood of collision. First, the ship’s possible safe anti-collision maneuvers are determined, and then the optimal maneuver is selected. Collision risk, represented by a domain or mathematical relationship, is used as a measure of safety. The optimality criterion for a safe maneuver is the additional time or distance spent executing the anti-collision maneuver, which contributes to the final deviation of the safe trajectory from the planned voyage route.

In this section, two safe control methods based on artificial intelligence and game theory, developed by the author of this article, are presented.

### 3.1. Control Task

A functional diagram of the control process, extending from remote sensing to secure control implementation, is shown in Figure 1.

In multi-ship navigation situations, controlling the subject ship requires selecting the optimal trajectory among possible safe paths using artificial intelligence and game theory tools. This first requires formulating a mathematical model of the control process and then applying an appropriate optimization technique [22]. The effectiveness of optimization depends on how well the model reflects the actual kinematics and dynamics of the control process. In this study, navigational safety is quantified either by the size of the passing ship domains generated via a neural network or by predicting the collision risk using an appropriate mathematical model. Both the kinematic and dynamic characteristics of the ships, as well as their degrees of cooperation, are taken into account.

### 3.2. Neural-Dynamic Control

The mathematical description of the control process includes the kinematic and dynamic equations of the subject ship and the dynamic constraints assigned to each encountered ship *j*. The kinematics of the motion of the subject ship relative to other ships are schematically presented in Figure 2.

The dynamics of the subject ship as an astatic object controlling the change in its course ψ_0_, with time constant *T*_ψ_ and gain *k*_ψ_, are represented by the following nonlinear differential equation:

(1) $$T_{\psi }{\ddot{\psi }}_{0}+a_{2}{\dot{\psi }}_{0}^{2}+a_{1}{\dot{\psi }}_{0}=k_{\psi }\alpha_{0}$$

where α is the rudder angle, while (*a*_1_, *a*_2_) are the coefficients of the nonlinear static of the subject ship’s characteristic ${\dot{\psi }}_{0}=f\left(\alpha \right)$.

The speed control dynamics *V*_0_ for the subject ship are described via a second-order static inertial system, with time constants *T_v_*_1_ and *T_v_*_2_ and a gain *k_v_*, reflecting the inertial effects during speed changes in anti-collision maneuvers, as follows:

(2) $$T_{v1}T_{v2}{\ddot{V}}_{0}+\left(T_{v1}+T_{v2}\right){\dot{V}}_{0}+V_{0}=k_{v}n_{0}$$

where *n*_0_ represents the rotational speed of the ship’s own propulsion system.

The model of the entire process includes the dynamics of maneuvering the subject ship (changes in course ψ_0_ and speed *V*_0_) and the kinematics of the passing ships, which move at a constant course ψ*_j_* and constant speed *V_j_*. The motion of the passing ships is taken into account in the constraints of the control process via mobile perilous domains generated using an artificial neural network.

In this approach, the following state equations and constraints of the NDC model are obtained:

(3) $${\dot{x}}_{1}=x_{5}\sin x_{3}\;{\dot{x}}_{2}=x_{5}\cos x_{3}\;{\dot{x}}_{3}=x_{4}\;{\dot{x}}_{4}=-\frac{a_{2}}{T_{\psi }}x_{3}^{2}-\frac{a_{1}}{T_{\psi }}x_{5}+\frac{k_{\psi }}{T_{\psi }}u_{01}\;{\dot{x}}_{5}=x_{6}\;{\dot{x}}_{6}=-\frac{T_{v1}{+T}_{v2}}{T_{v1}T_{v2}}x_{6}-\frac{1}{T_{v1}T_{v2}}x_{5}+\frac{k_{v}}{T_{v1}T_{v2}}u_{02}$$

where, for the subject ship’s state and control variables, (*x*_1_, *x*_2_) = (*x*_0_, *y*_0_) are the position coordinates, *x*_3_ = ψ_0_ is the heading, *x*_4_ = $\dot{\psi_{0}}$ is the angular velocity of the course change, *x*_5_ = *V*_0_ is the velocity, *x*_6_ = $\dot{V_{0}}$ is the acceleration, *u*_01_ = α_0_ is the rudder deflection, and *u*_02_ = *n*_0_ is the propeller speed.

The transport process constraints *C* are defined as follows:

(4) $$C\left(x_{3},x_{5},\psi_{j},V_{j},\varphi_{j},D_{j},T_{s},D_{s}\right)\geq 0$$

where φ*_j_* and *D_j_* denote the bearing to and distance from ship *j*, respectively, and *T_s_* and *D_s_* represent the safe time and distance for the subject ship relative to the approaching ship *j*.

To implement constraint (4) of the control process—namely the safe passing of the subject ship by ship *j*—an artificial neural network was used to estimate the collision risk *r_j_*, and the sizes of the dangerous maneuver domains assigned to the encountered ships were adjusted.

Domains in the shape of a circle, hexagon, ellipse, or parabola, with size proportional to the collision risk *r_j_*, are generated by a neural network from the MATLAB 2026 Deep Learning Toolbox (Figure 3).

For a stationary object, the domain will be a circle with a radius equal to the safe overtaking distance *D_s_*. For a moving object, the domain shape can be chosen to incorporate the COLREG right-of-way rules. These apply separately for good and restricted visibility conditions. In good visibility conditions, the subject ship must yield to object *j* on the right. However, if you encounter ship *j* coming from the left, you are expected to yield to your right-of-way. In restricted visibility conditions, the right-of-way rule on the right does not apply; only an increased safe overtaking distance *D_s_* applies. In such cases, the overshoot area is assumed to be a circle with a radius *D_s_*.

The basic domain sizes are described by the following relations:

- Circular domain:

(5) $$g_{j}^{circle}\left(X_{j},\;Y_{j}\right)=X_{j}^{2}+Y_{j}^{2}-D_{s}^{2}\geq 0$$

where (*X_j_, Y_j_*) are the coordinates of the distance *D_j_* of ship *j* from the subject ship.

- Hexagonal domain:

(6) $$L_{dj}=1.1L_{j}\left(1+0.345V_{j}^{1.6}\right)$$

(7) $$W_{dj}=1.1\left(W_{j}+0.767L_{j}V_{j}^{0.4}\right)$$

where *L_j_* and *W_j_* are the length and width of ship *j*, and *V_j_* is the speed of ship *j*.

- Ellipsoidal domain:

(8) $$g_{j}^{ellipse}\left(X_{j},\;Y_{j}\right)={\left(X_{j}\sin\psi_{j}+Y_{j}\cos\psi_{j}-F_{j}\right)}^{2}W_{dj}^{2}+{\left(X_{j}\cos\psi_{j}+Y_{j}\sin\psi_{j}-F_{j}\right)}^{2}L_{dj}^{2}-L_{dj}^{2}W_{dj}^{2}\geq 0$$

where *F_j_* is the focal distance of the ellipse.

- Parabolic domain:

(9) $$g_{j}^{parabola}\left(X_{j},\;Y_{j}\right)=X_{j}\sin\psi_{j}+Y_{j}\cos\psi_{j}-S{\left(X_{j}\cos\psi_{j}+Y_{j}\sin\psi_{j}\right)}^{2}\geq 0$$

where *S* is the span of the parabola arms.

The task of the neural network, with its six inputs and one output, is to map *R* the collision risk *r_j_* by dividing the set of possible navigation situations when the subject ship passes the encountered ship *j* into five subsets, describing the situation as “safe” for *r_j_* = 0.1; “warning” for *r_j_* = 0.3; “possible risk” for *r_j_* = 0.5; “dangerous” for *r_j_* = 0.7; and “collision” for *r_j_* = 0.9, and is given as follows:

(10) $$r_{j}=R\left(V_{0},\;\psi_{0},\;V_{j},\;\psi_{j},\;D_{j},\;\varphi_{j}\right)$$

where *r_j_* is the network response, *R* is the mapping demonstrated by the network, *V*_0_ and ψ_0_ represent the subject ship’s speed and course, *V_j_* and ψ*_j_* are the encountered ship *j*’s speed and course, and *D_j_* and φ*_j_* are the distance and bearing to ship *j* (Figure 4).

To reflect the navigator’s subjective maneuvering decisions, the domain sizes change over the course of ship *j*’s motion process proportional to the collision risk.

The neural network, with its single output *r_j_*, was previously trained by a group of experienced navigators using several standard scenarios of navigation situations at sea. For each situation, the navigator selected the best option from a subset based on their own judgment, that is, subjectively, in accordance with good seamanship. In doing so, the trained network represents the average experience of a larger group of navigators. Figure 5 shows an example of the network’s error function during training.

Simulation scenarios used in ARPA (automatic radar plotting aid) courses—designed to teach radar operation and automatic target tracking under conditions closely mirroring real-world maritime environments—served as the training scenarios:

- Anti-collision scenarios compliant with COLREGs (head-on encounters, crossing courses, overtaking).
- Trial maneuver scenarios (avoiding multiple targets simultaneously—multi-threat simulation).
- Restricted visibility scenarios (navigation based solely on radar readings, clutter filtering).
- Navigation scenarios in challenging and congested areas (Traffic Separation Schemes—TSS, pilotage navigation, and Parallel Indexing techniques).
- Emergency and system failure scenarios (lost target, target swap, power or sensor failure).

The network was trained by 175 navigators during ARPA training courses, and the data included the so-called radar report, comprising the following parameters characterizing vessel movement: speed and course, range and bearing, DCPA and TCPA, and aspect angle. Classification accuracy depended on the accuracy of data acquisition from the radar, speed log, and gyrocompass. The combined effect of errors on the quality of the navigational situation display must not exceed ±1% for absolute values and ± 0.5° for angular quantities. The scenarios used to train the network were not used for the control studies presented in the paper. Separate scenarios were prepared for testing the neural-dynamic and game-based control algorithms, specifically regarding the number of passing vessels. The number of training samples was 1860.

A neural network represents heuristic knowledge similar to that of an experienced navigator. The accuracy of a neural network’s assessment of the safety of passing ships depends largely on the accuracy of the patterns used in the network’s learning process. Expanding the knowledge of experienced navigators could lead to the network exhibiting better “average” knowledge (Figure 6).

The neural network first calculates the current collision risk index *r_j_* [0, 1], and its value serves as a scaling factor for the geometric size of the base domain *R_base_*. For the ship domain types employed—whose basic dimensional parameters are shown in Figure 3—their dynamic values (*L_dj_*, *W_dj_*) are dynamically scaled using nonlinear sigmoidal logistic functions. Since the neural network in use naturally employs a sigmoid activation function, the following modified logistic function is applied to map the domain size *R*:

(11) $$R\left(r_{j}\right)=R_{base}+\frac{R_{max}-R_{base}}{1+e^{-\lambda \left(r_{j}-r_{j0}\right)}}$$

where *R_base_* is the minimum safe domain size under zero-risk conditions, *R_max_* is the maximum permissible domain size representing the boundary of the early-warning zone for COLREG compliant maneuvers, λ is the curve slope coefficient representing the algorithm’s sensitivity, and *r_j_*_0_ denotes the threshold for transitioning into the intensive maneuvering phase—for example, *r_j_*_0_ = 0.5.

The domain dimensions are scaled according to the network output, which represents the collision risk; these domains are assigned to individual passing ships and move at their speeds in the dynamic programming plane [23,24].

Among the real-time dynamic optimization techniques, dynamic programming proved to be the most suitable option for modeling the considered control process defined by the state (3) and time-dependent state constraints (4). Since collision avoidance satisfied the duality conditions, the time-optimal path of the subject ship was derived based on Bellman’s principle, proceeding from stage *k* = 1 to stage *K*. The state equations in discrete forms are defined as follows:

(12) $$x_{1,k+1}=x_{1,k}+x_{5,k}∆t_{k+1}\sin x_{3,k}\;x_{2,k+1}=x_{2,k}+x_{5,k}∆t_{k+1}\sin x_{3,k}\;x_{3,k+1}=x_{3,k}+x_{4,k}∆t_{k+1}\;x_{4,k+1}=x_{4,k}-\frac{a_{2}}{T_{\psi }}x_{4,k}\left|x_{4,k}\right|-\frac{a_{1}}{T_{\psi }}x_{4,k}+\frac{k_{\psi }}{T_{\psi }}u_{01}\;x_{5,k+1}=x_{5,k}+x_{6,k}∆t_{k+1}\;x_{6,k+1}=x_{6,k}-\frac{T_{v1}+T_{v2}}{T_{v1}T_{v2}}x_{6,k}-\frac{1}{T_{v1}T_{v2}}x_{5,k}+\frac{k_{v}}{T_{v1}T_{v2}}u_{02}$$

According to Bellman’s principle, the time-optimal control of the subject ship at stage k of the trajectory is given by the following formula:

(13) $$t_{k}^{*}=\underset{\begin{array}{c} u_{1,k-2}\\ u_{2,k-2} \end{array}}{\min}\left[t_{k-1}^{*}\left(x_{1,k},x_{2,k},x_{3,k-1},x_{4,k-1},x_{5,k-1},x_{6,k-1}\right)+∆t_{k}\left(x_{1,k},x_{2,k},x_{1,k+1},x_{2,k+1},x_{5,k}\right)\right],k=1,\ldots ,K$$

The NDC method uses the state description of the control process (5) to model the dynamic motion of the subject ship, and (6) determines its time-optimal control, taking into account the kinematics of the passing ships, represented by their neural domains.

The dynamic programming algorithm is multi-stage, and the process state constraints, in the form of domains assigned to passing ships, are refreshed in the next stage of calculating the safe trajectory of the subject ship.

According to the Nyquist–Shannon sampling theorem, the discrete time step Δ*t_k_* in Equation (12) was set to be approximately an order of magnitude smaller than the time constant Tψ of the tested ship 0 (i.e., from 1 s to 30 s), depending on the ship’s tonnage. The state-space grid resolution is constant and depends on the duration of the test ship’s course change maneuvers, which, according to the control theory, is approximately three times the constant *T*_ψ_ and is then converted to navigational distance. Consequently, the dynamic programming grid resolution ranges from 0.1 nautical mile to 1.0 nautical mile.

### 3.3. Game Control

A multi-stage matrix game was constructed as a matrix ***R*** containing collision risk values *r_j_* for permissible course or speed changes for the subject ship (rows) as *n* strategies *s*_0,*n*_ and other ships (*j*) (columns) as *m* strategies *s_j,m_*:

(14) $$R_{j}\left[r_{j}\left(r_{s_{0,n}},r_{s_{j,m}}\right)\right]=\left[\begin{array}{c} r_{s_{0,1}{,s}_{j,1}},\;\ldots ,r_{s_{0,1}{,s}_{j,m}},\;\ldots ,\;r_{s_{0,1}{,s}_{j,M}}\\ r_{s_{0,2}{,s}_{j,1}},\;\ldots ,\;r_{s_{0,2},s_{j,m}},\;\ldots ,r_{s_{0,2},s_{j,M}}\\ \ldots \\ r_{{s_{0,n},s}_{j,1}},\ldots ,\;r_{s_{0,n},s_{j,m}},\;\ldots ,r_{s_{0,n},s_{j,M}}\\ \ldots \\ r_{s_{0,N},s_{j,1}},\ldots ,\;r_{s_{0,N},s_{jm}},\;\ldots ,r_{s_{0,N},s_{j,M}}\\ \; \end{array}\right]$$

where *n* = 1, 2, …, *N* denotes the number of strategies for the subject ship, and *m* = 1, 2, …, *M* denotes strategies for the other ships.

Many mathematical formulas are available for estimating collision risk [25,26,27,28,29,30]. Ship collision risk models include both probabilistic, physics-based methods and advanced machine learning algorithms that consider ship attributes and navigational and environmental factors. The presented models quantify the risk of collision *r_j_* using parameters such as DCPA = $D_{min}^{j}$ and TCPA = $T_{min}^{j}$.

In this article, the author proposes the following formulas: logarithmic (*log*), mean square (*ms*), and hyperbolic (*hyp*).

The logarithmic model *log* calculates the risk of collision *r_j_* between the subject ship and ship *j* using a logarithmic function that emphasizes the distance between ships *D_j_*, especially in situations of excessive proximity:

(15) $$r_{j}^{log}=\left(1-0.1\;log\left(\frac{D_{min}^{j}}{D_{s}}\right)\right)\left(1-0.2\frac{T_{min}^{j}}{T_{s}}\right)+{\left(\frac{D_{s}}{D_{j}}\right)}^{2}-0.2$$

where $D_{min}^{j}\;and\;T_{min}^{j}$ denote the distance and time to closest approach between the subject ship and the encountered ship *j*, while *D_s_* and *T_s_* are predetermined safe distance and approach time values for the subject ship and ship *j* in the actual navigational situation.

In the root-mean-square model *ms*, the risk of collision *r_j_* is represented as the reciprocal of the square root of the average of the squares for the individual quantities of $D_{min}^{j}/D_{s}$ and $T_{min}^{j}/T_{s}$ to closest approach and relative proximity $D_{s}/D_{j}$:

(16) $$r_{j}^{ms}={\left({\left(\frac{D_{min}^{j}}{D_{s}}\right)}^{2}+{\left(\frac{T_{min}^{j}}{T_{s}}\right)}^{2}+{\left(\frac{D_{s}}{D_{j}}\right)}^{2}\right)}^{-\frac{1}{2}}$$

In the hyperbolic model *hyp*, the collision risk *r_j_* with ship *j* is inversely proportional to the relative distance $D_{min}^{j}/D_{s}$ and time $T_{min}^{j}/T_{s}$ values:

(17) $$r_{j}^{hyp}=0.5{\left(\frac{D_{min}^{j}}{D_{s}}\right)}^{-1}+0.5{\left(\frac{T_{min}^{j}}{T_{s}}\right)}^{-1}+0.2$$

The collision risk graphs presented in Figure 7 for individual models were created using Matlab 2026 software [31].

The task of safely steering the subject ship while passing *j* other ships is considered as a multi-player matrix game with a finite number of players and a finite number of strategies, in which a Nash equilibrium exists in multiple mixed strategies. In such situations, choosing the optimal strategy becomes a decision problem. The concept of entropy, most often Shannon entropy, is then introduced into these games as the choice of the strategy with the lowest risk when the player does not have full information about the opponents’ intentions.

As the assumed matrix game does not have a saddle point in real navigational situations, its solution is carried out in mixed strategies, expressed as probability distributions $P\left(p_{s_{0,n}},\;p_{s_{j,m}}\right)$ of the pure strategies $s_{j,m}$ of the subject ship and the *J* passed ships. To aggregate the strategies of the subject ship over all *J* ships, the entropy *H* was used to assess the certainty of the components of the pure strategies:

(18) $$H\left(P_{s_{0,n},s_{j,m}}\right)=-\sum_{s_{j,1}}^{s_{j,M}}P_{s_{0,n}{,s}_{j,m}}{\;log\;P}_{s_{0,n}{,s}_{j,m}}$$

The optimal strategy for the subject ship then satisfies a minimum-entropy criterion [32,33]:

(19) $$u_{0}^{*}=u_{{0,s}_{0}}\left(H_{min}\right)$$

The statement that minimum entropy is equivalent to minimum risk refers to the profound connection between information theory (entropy) and decision theory or statistics (Bayesian risk) [34]. In the context of learning systems and classification, minimizing the entropy of the output distribution directly leads to minimizing the risk of making an incorrect decision. The mathematical bridge connecting these two realms is Fano’s inequality, which demonstrates that the minimum error incurred when guessing a random variable is strictly bounded by that variable’s conditional entropy:

(20) $$H\left(x|y\right)\leq H_{b}\left(P_{e}\right)+P_{e}{\;\log}_{2}\left(P_{e}\left|x\right|-1\right)$$

where *H*(*x*|*y*) is the conditional entropy of variable *x* given, representing the uncertainty remaining about *x* after observing *y*; *P_e_* is the probability of error *e*; *H_b_*(*P_e_*) is the binary entropy function, defined as *H_b_*(*P_e_*) = −*P_e_* log_2_(*P_e_*) − (1 − *P_e_*) log_2_(1 − *P_e_*); and |*x*| is the number of all possible values that variable *x* can take.

The mixed-strategy Nash equilibrium acts as a minimax solution, meaning that the expected payoff in the equilibrium guarantees each player the optimal outcome against the most hostile, rational opponent [35,36].

The cooperative game control method, termed GCc, is based on dual linear programming. Finding the optimal strategy for the subject ship $u_{0{,s}_{0}}$, with strategy probabilities $p_{s_{0}}$, minimizes the risk of collision with ship *j*,

(21) $$\underset{u_{0{,s}_{0}}}{\min}\;r_{j}$$

with the conditions

(22) $$-\sum_{s_{0,1}}^{s_{0,N}}r_{s_{0,n,},s_{j,m}}P_{s_{0,n}}+r_{j}\geq 0$$

(23) $$\sum_{s_{0,1}}^{s_{0,N}}P_{s_{0,n}}=1$$

and the cooperating ship encountered also minimizes the risk of collision:

(24) $$\underset{u_{j,s_{j}}}{\min}\;r_{j}$$

However, in the absence of cooperation, the non-cooperative control method, GCnc, accounts for navigational situations where an encountered vessel *j* poses a collision risk—whether due to a failure to yield right-of-way to another ship in accordance with COLREGs, constituting passive non-cooperation, or as a result of a misjudgment of the situation,

(25) $$\underset{u_{j,s_{j}}}{\max}\;r_{j}$$

with the conditions

(26) $$-\sum_{s_{j,1}}^{s_{j,M}}r_{s_{0,n,},s_{j,m}}P_{s_{j,m}}+r_{j}\geq 0$$

(27) $$\sum_{\psi_{j,1}}^{\psi_{j,M}}P_{s_{j,m}}=1$$

In summary, the game control method uses the state equations of ship’s motion kinematics; the optimal strategy for the subject ship (19) and the two-criteria game optimization involves using either Formulas (21) and (24) or Formulas (21) and (25).

### 3.4. Basic Safe Control

Basic safe control (BSC) is a commonly used method when using the anti-collision ARPA system. It involves determining a sequence of necessary course or speed changes for the subject ship to avoid collision, assuming a safe passing distance *D_s_*, derived from the COLREG rules. It is assumed that each encountered ship *j* is traveling at a constant heading ψ*_j_* and speed *V_j_*. The basis for BSC algorithm synthesis is to establish a set of permissible changes in course ψ_0_ and speed *V_j_* for the subject ship that will ensure the safe passing of the *j* encountered ship (Figure 8).

The optimal basic safe control ${\vec{V_{0}}}^{*}$ of ship 0 in relation to all passing *j* ships is determined from the joint set of permissible maneuvers by a single linear programming algorithm:

(28) $$\underset{{\vec{V_{0}}}^{*}}{\min}\;t_{0}=t_{0}^{*}\to d_{0}^{*}$$

where *t*_0_ is the time of passing all encountered *j* ships, and *d*_0_ is the final deviation of the safe trajectory.

### 3.5. Sensitivity of Safe Control

The sensitivities $s_{d}^{NDC}$ of NDC, $s_{d}^{GC}$ of GC, and $s_{d}^{BSC}$ of BSC are defined as the relation of the relative change in the final deviation *d* in the subject ship’s safe trajectory to the relative change in the safe passing distance *D_s_*:

(29) $$s_{d}^{NDC,GC,BSC}=\underset{∆D_{s}\to 0}{\lim}\frac{\frac{∆d}{d}}{\frac{∆D_{s}}{D_{s}}}=\underset{∆D_{s}\to 0}{\lim}\frac{D_{s}\cdot ∆d}{d\cdot ∆D_{s}}=\frac{D_{s}}{d}\frac{∆d}{∆D_{s}}$$

The aim of this paper was to synthesize and investigate neural-dynamic and game-based methods for the safe control of a ship during multi-object avoidance maneuvers. Safe ship control relies primarily on maintaining an appropriate *D_s_* safe passing distance. Consequently, the paper focuses on assessing the sensitivity of the safe control system—specifically regarding changes to the safe distance, the value of which is derived from COLREG requirements.

During the design of the NDC and GC algorithms, the values of the following control process model parameters were analyzed: neural network weight coefficients, collision risk model coefficients, grid resolution for the dynamic programming method, and the discrete step size for game strategies.

Specifically:

- Neural network weight coefficients are selected autonomously during network training.
- Collision risk model coefficients are determined by the need to scale them within the [0, 1] range.
- The dynamic programming grid resolution is directly determined by the need to match domain dimensions—which depend on the safe distance —with the resolution set to an order of magnitude smaller than *D_s_*.
- The discrete step size in game strategies is determined by COLREG requirements, ensuring that the maneuver is recognizable.

During the evaluation of these parameter values while investigating NDC and GC methods, it became evident that variations in these parameters had a minimal impact on the sensitivity of the safe control system.

The sensitivity *s_rj_* of collision risk is defined as the relationship between the changes in the risk of collision and the relative changes in the distance and time to excessive approach of the ships:

(30) $$s_{r_{j}}=\frac{\partial r_{j}\left(\frac{D_{min}^{j}}{D_{s}},\frac{T_{min}^{j}}{T_{s}}\right)}{\partial \left(\frac{D_{min}^{j}}{D_{s}},\frac{T_{min}^{j}}{T_{s}}\right)}=\underset{\frac{D_{\min}^{j}}{D_{s}},\frac{T_{\min}^{j}}{T_{s}}}{\mathrm{grad}}r_{j}\left(\frac{D_{min}^{j}}{D_{s}},\frac{T_{min}^{j}}{T_{s}}\right)=\frac{\partial r_{j}}{\partial \left(\frac{D_{min}^{j}}{D_{s}}\right)}\frac{\partial r_{j}}{\partial \left(\frac{T_{min}^{j}}{T_{s}}\right)}$$

For the logarithmic model *log*, the collision risk sensitivity is

(31) $$s_{r_{j}}^{log}=\frac{0.4w_{1}w_{2}}{\frac{D_{min}^{j}}{D_{s}}}\left(1-w_{1}log\frac{D_{min}^{j}}{D_{s}}\right)\left(1-w_{2}\frac{T_{min}^{j}}{T_{s}}\right)$$

For the mean-square model *ms*, the collision risk sensitivity is

(32) $$s_{r_{j}}^{ms}=\frac{16w_{1}w_{2}\frac{D_{min}^{j}}{D_{s}}\frac{T_{min}^{j}}{T_{s}}}{w_{1}{\left(\frac{D_{min}^{j}}{D_{s}}\right)}^{2}+w_{2}{\left(\frac{T_{min}^{j}}{T_{s}}\right)}^{2}}$$

For the hyperbolic model *hyp*, the collision risk sensitivity is

(33) $$s_{r_{j}}^{hyp}=\frac{w_{1}w_{2}}{{\left(\frac{D_{min}^{j}}{D_{s}}\right)}^{2}{\left(\frac{T_{min}^{j}}{T_{s}}\right)}^{2}}$$

The sensitivity characteristics of collision risk according to relative changes in safe distance and approach time for the *log*, *ms*, and *hyp* models are presented in Figure 9.

From these sensitivity plots, it can be seen that when distances and times approach their safe thresholds, the hyperbolic model *hyp* exhibits the greatest sensitivity in tight-proximity scenarios, while the logarithmic model *log* shows the least sensitivity.

## 4. Experimental Results

A comparative evaluation of the NDC and GC methods for the safe control of the subject ship was conducted in situations with varying numbers of passing ships. The simulation studies were based on actual navigational situations recorded on the research and training vessel r/v HORYZONT II: L = 56.34 m; B = 11.36 m; T = 5.3 m; DWT = 396 t [37]. The navigational situations were recorded on the SAM Electronics ARPA radar in the Skagerrak and Kattegat Straits in September 2025 during a training and research cruise to Spitsbergen (Figure 10).

Simulation tests were performed on navigational situations involving the subject ship passing *J* = 1, 3, and 12 ships, whose motion parameters are presented in Table 1.

Figure 11 visualizes the studied navigational situations in the form of ship velocity vectors.

### 4.1. Computer Simulation of the NDC Algorithm

A summary of the test results for the neural-dynamic control method, NDC, and the basic safe control method, BSC, is presented in Table 2.

The computation times presented in Table 2 confirm the advantage of the dynamic programming approach used in the NDC method: the greater the number of process state constraints in the form of moving domains, the fewer admissible grid nodes remain for determining the ship’s optimal safe trajectory, and the shorter the computation time.

Figure 12, Figure 13 and Figure 14 show the results of computer simulations of the safe trajectories of the subject ship when passing one, three, and 12 other ships, conducted under conditions of good visibility at sea at *D_s_* = 1 nm and restricted visibility at *D_s_* = 3 nm; the NDC method establishes constraints on the process state, represented by a hexagon NDC_h, a parabola NDC_p, and an ellipse NDC_e.

A ship’s safe and optimal course depends on the prevailing navigation conditions. According to COLREG regulations, this is assessed based on the safe passing distance *D_s_*. Based on the experience of ship crews and experimental studies, the safe passing distance *D_s_* ranges from approximately 0.5 nautical miles in good visibility to 3.0 nautical miles in restricted visibility. The characteristics of the final deviation *d* in the safe subject ship’s trajectory, determined by the NDC method, depending on the safe passing distance *D_s_*, were compared with the characteristics calculated using the basic safe control method (BSC) (Figure 15).

The neural-dynamic control method of NDC_h, with hexagonal domains when passing *J* ships, exhibits the largest final deviation *d* in the subject ship’s safe trajectory, and the smallest with ellipsoidal domains. This deviation, compared to the BSC method, is larger for a small number of passing ships and comparable for a larger number of passing ships.

Figure 16 illustrates the sensitivity attributes of ship safe control in response to variations in navigation conditions. These characteristics are derived from the neural-dynamic control methods utilizing ship domains, represented as hexagons—NDC_h, parabolas—NDC_p, and ellipses—NDC_e, and, for comparison, by performing an assessment according to the base safe control method—BSC. The sensitivity values of safe control were calculated via Equation (26) for the graphs shown in Figure 15.

The neural-dynamic control method with hexagonal domains, NDC_h, for *J* passing ships shows the highest sensitivity, whereas with ellipsoidal domains, NDC_e shows the lowest sensitivity. However, the sensitivity is comparable for small safe distance values *D_s_* regardless of the number of passing ships.

### 4.2. Computer Simulation of the GC Algorithm

The GC field control method presented in Section 3.3 enabled simulation experiments to be conducted in conditions of cooperation and non-cooperation between the subject ship and ships *j* to avoid collisions. Figure 17 presents the ship trajectories determined via the cooperative field control method, GCc_log, and the non-cooperative field control method, GCnc_log, with a logarithmic collision risk *r_j_* model, in a situation in which the subject ship passed *J* = 3 ships.

A summary of the test results for the cooperative (GC_c) and non-cooperative (GC_nc) game control algorithms is presented in Table 3.

Figure 18, Figure 19 and Figure 20 show the simulation results for the subject ship’s safe trajectories in situations in which it passed *J* = 1, 3, and 12 ships, both in conditions of good visibility at *D_s_* = 1 nm and restricted visibility at *D_s_* = 3 nm, determined via the cooperative and non-cooperative GC methods, taking into account three collision risk models *r_j_*: logarithmic *log*, mean square *ms*, and hyperbolic *hyp*.

The GC_log method with a logarithmic collision risk model shows the smallest final deviation *d* in the subject ship’s safe trajectory from the reference route, while the GC_hyp method with a hyperbolic model shows the largest final deviation. Tripling the safe passing distance *D_s_* due to restricted visibility at sea doubles the deviation in the subject ship’s trajectory from the predetermined route. The GCnc method, on the other hand, increases the deviation *d* in the trajectory compared to the GCc algorithm by 30–50%.

The quality of the safe game control is characterized by the value *d* of the final deviation in the subject ship’s safe trajectory from the reference voyage route, depending on the navigational situation, related to the value of the safe passing distance *D_s_* resulting from the COLREG requirements. Figure 21 shows a comparison of the deviation characteristics for game control and non-game control.

Figure 22 shows the sensitivity characteristics of the safe control of the subject ship to changes in navigation conditions, corresponding to the following collision risk models: logarithmic GC_log, mean square GC_ms, and hyperbolic GC_hyp. The sensitivity values for safe control were calculated using Equation (26) for the graphs shown in Figure 21.

## 5. Discussion

The collision risk sensitivity characteristics shown in Figure 9—which exhibit varying degrees of linearity or nonlinearity—reflect potential ship close-quarters situations, depending on the mathematical risk model adopted. This is particularly evident in close-quarters situations where $\frac{D_{min}^{j}}{D_{s}}=1$ but where the timing falls either near the $\frac{T_{min}^{j}}{T_{s}}=1$ mark or far out at $\frac{T_{min}^{j}}{T_{s}}=10$.

Research findings regarding the optimality of safe ship trajectories—analyzed in terms of navigational situation complexity involving varying numbers of passing vessels—indicate the value of extending this study to scenarios involving larger numbers of vessels (up to several dozen) in areas of heavy traffic. In such cases, the sensitivity of safe control increases from approximately 70% to 180%.

A comparison of the optimality graphs for safe ship trajectories in Figure 15 and Figure 21 reveals that the number of passing objects has a different impact depending on whether neural-dynamic control or game-based control is employed.

In practical application, the measure of the effectiveness of safe ship control using NDC and GC methods can be defined as the relative sensitivity metric,

(34) $$s_{0}=\left|\frac{s_{d}^{NDC,GC}-s_{d}^{BSC}}{s_{d}^{BSC}}\right|100\%$$

where the values of $s_{d}^{NDC,GC}$ and $s_{d}^{BSC}$ are obtained from Formula (29) [38,39].

The characteristics of the effectiveness of the safe control of the subject ship *s*_0_, based on the simulation results of the methods that form the bases of Figure 16 and Figure 22, are presented in Figure 23.

In good visibility conditions at sea and with lower required safe passing distances *D_s_*, the GCnc method is most effective at 80%. As navigational conditions deteriorate and *D_s_* values need to be increased, the GCc method is most effective at 60%, followed by the NDC method to a lesser extent (35%).

Despite these favorable findings, this study has several limitations. The NDC method is extremely sensitive to the shape of the passing ship domains, and the GC method is highly dependent on the form of the mathematical collision risk model.

## 6. Conclusions

Simulation studies demonstrated the correct operation of safe ship control algorithms in complex multi-ship traffic situations by taking into account environmental visibility constraints and the level of cooperation between ships resulting from the interpretation of COLREG traffic priority rules. The effectiveness of the NDC method, which utilizes artificial intelligence elements in the form of an artificial neural network that assigns collision risk domains to ships, was compared with the effectiveness of the GC method, which utilizes game-theoretic elements that consider ship cooperation—or lack thereof—in avoiding collisions.

Compared to previous studies, the novelty of this study is the joint application of neural network methods and game control under realistic navigation scenarios, with both good and restricted visibility at sea [5,8,10,21,23,30].

The methodology used in this study shows how AI and game theory can tackle the following practical aspects of safe navigation:

- Safe passing in multi-ship traffic situations;
- The coordination of ships to ensure collision avoidance;
- The impact of the current navigational situation on navigational safety.

As the NDC and GC methods enable the live generation of neural domains and ship equilibrium states in risk games, they provide more realistic representations of control mechanisms, making them a promising approach for improving maritime safety.

Future research should:

- Investigate the sensitivity of safe ship control methods to inaccuracies in sensors (radar, log, and gyrocompass) monitoring the navigational situation;
- Analyze the sensitivity of ship controls to changes in the adopted parameters of the neural network mathematical models (structure, weight coefficients, and activation function) and collision risk models (function type and weight coefficients);
- Incorporate varying resolutions of the dynamic programming grid and discrete step sizes of the game strategy into the research;
- Develop a method for visualizing safe ship trajectories on the display of an ARPA anti-collision system.

## Figures and Tables

**Figure 1:**
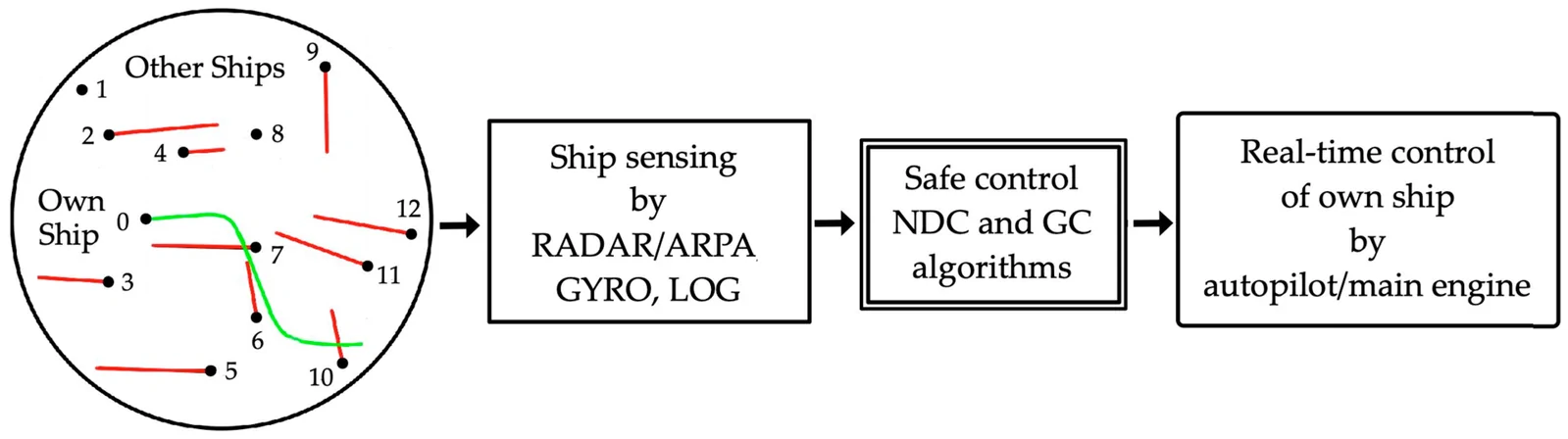
A flow chart showing safe ship control in multi-ship situations, illustrated in the form of their six-minute velocity vectors. RADAR = radio detection and ranging; ARPA = automatic radar plotting aid; GYRO = gyrocompass; LOG = speed measurement; NDC = neural-dynamic control; GC = game control.

**Figure 2:**
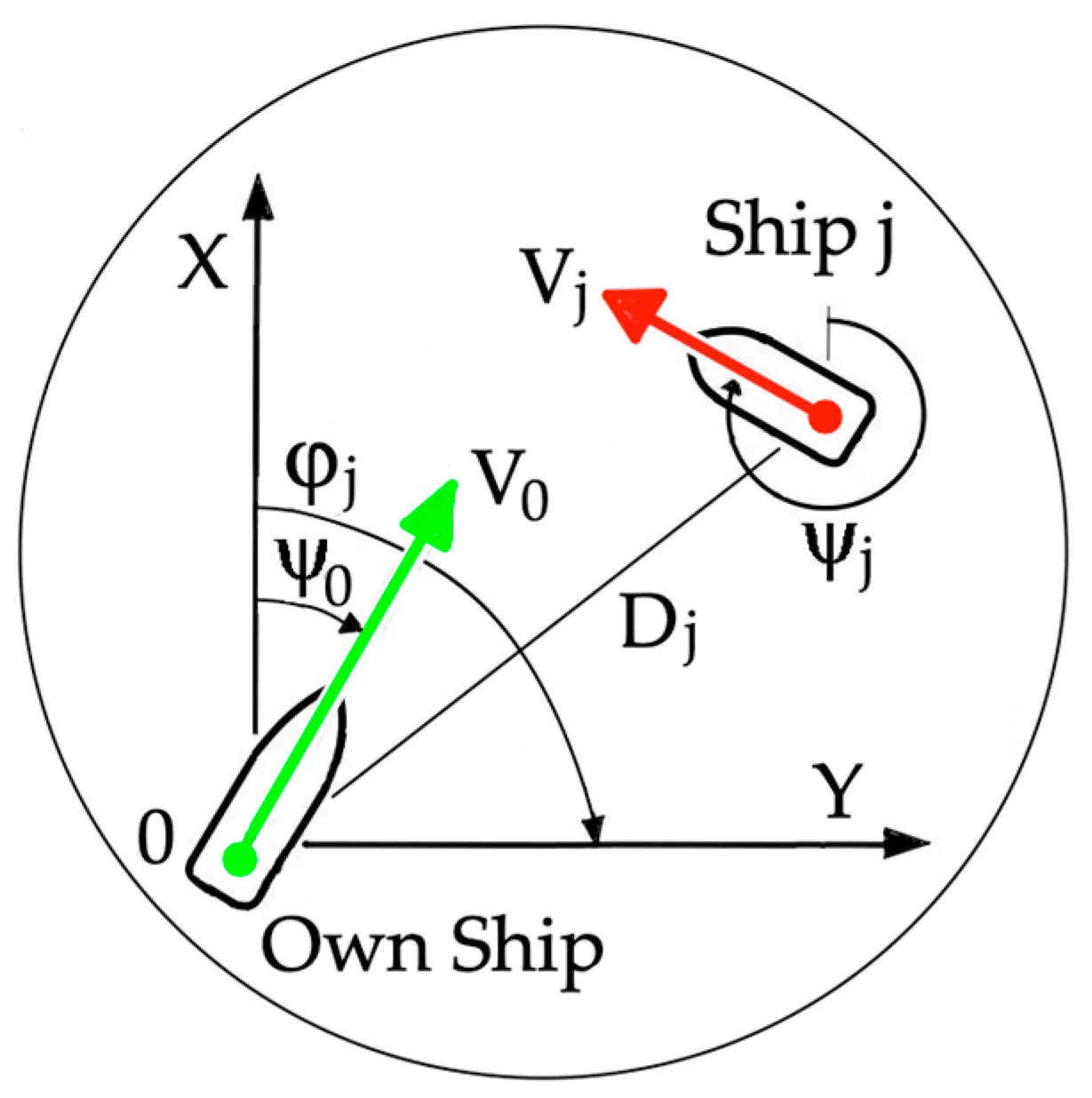
Illustration of a control task demonstrating the safe and optimal trajectory of the subject ship and the relative motion of ship *j* in relation to the subject ship in an (*X*, *Y*) coordinate system. (ψ_0_, *V*_0_) represents the course and speed of the subject ship; (ψ*_j_*, *V_j_*) represents the course and speed of ship *j*; and (φ*_j_*, *D_j_*) represents the bearing of and distance to ship *j*.

**Figure 3:**
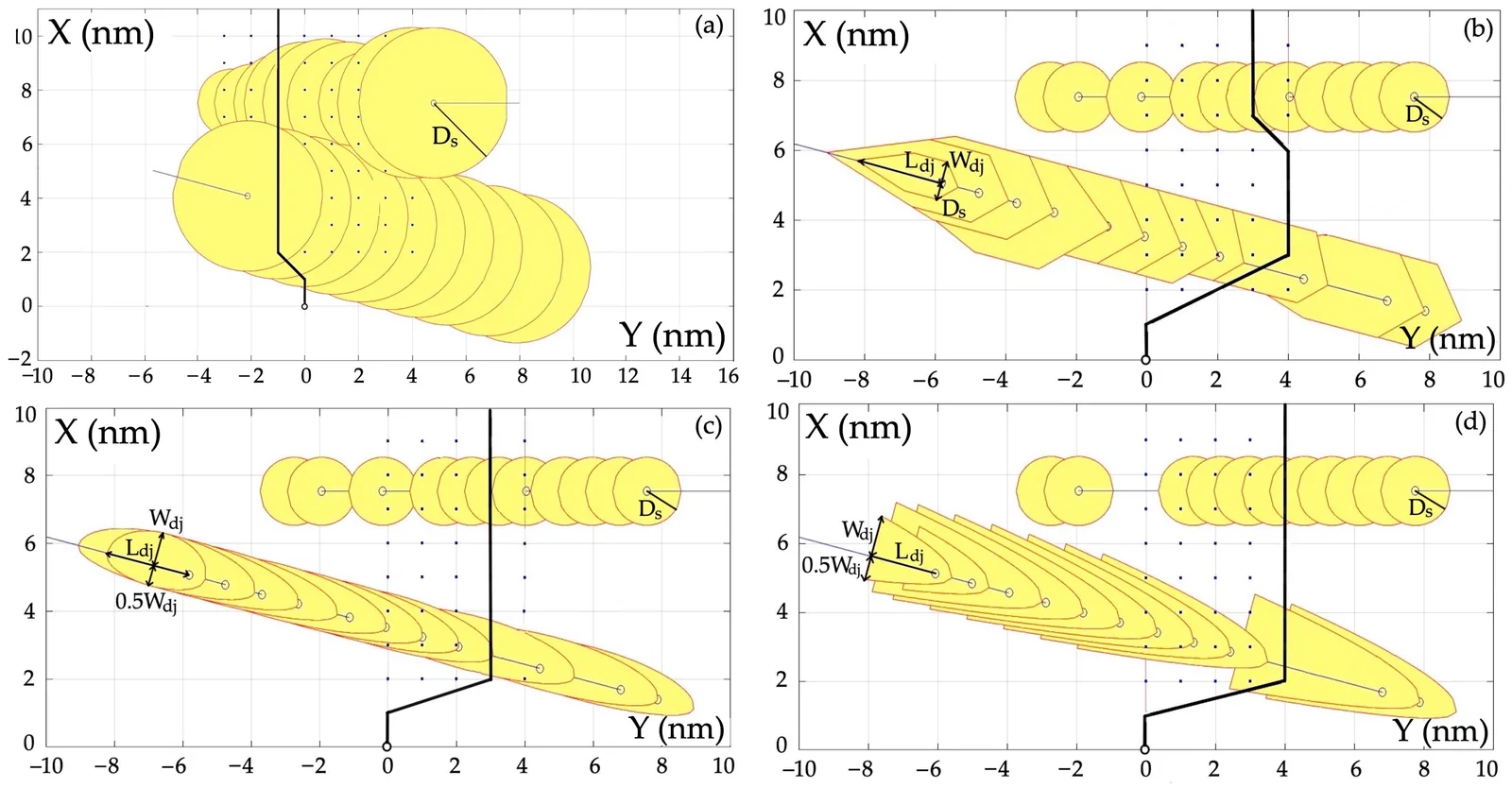
Control process constraints in the form of domains: (**a**) circular, (**b**) hexagonal and circular, (**c**) ellipsoidal and circular, and (**d**) parabolic and circular.

**Figure 4:**
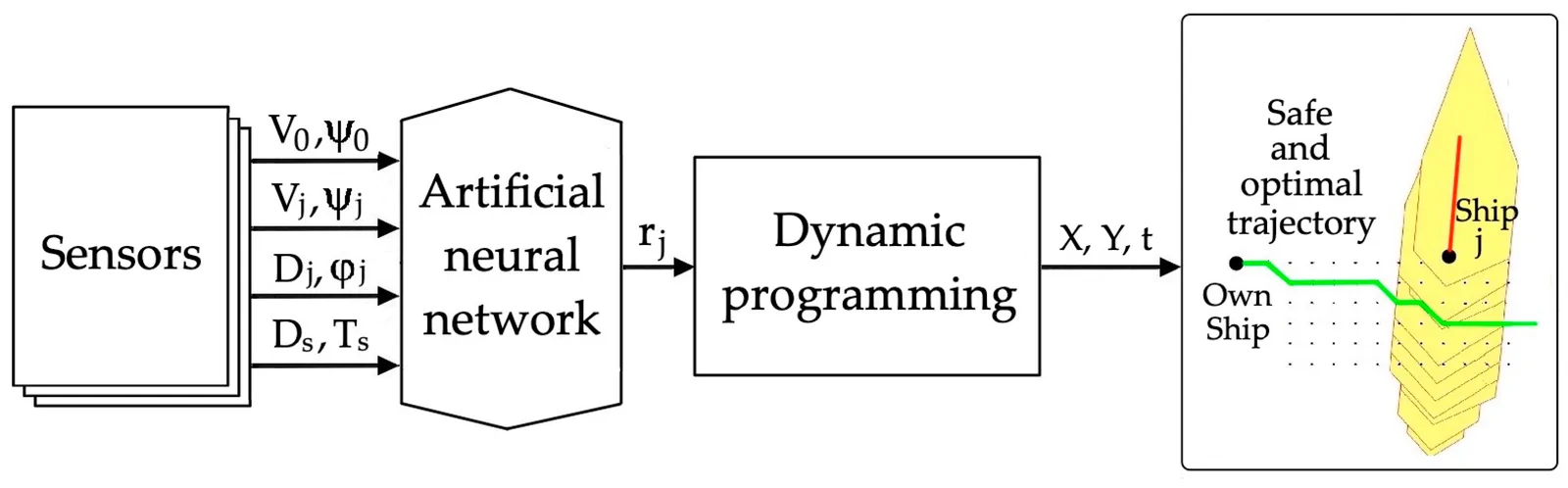
Generating constraints that depict the collision risk *r_j_* through a neural network.

**Figure 5:**
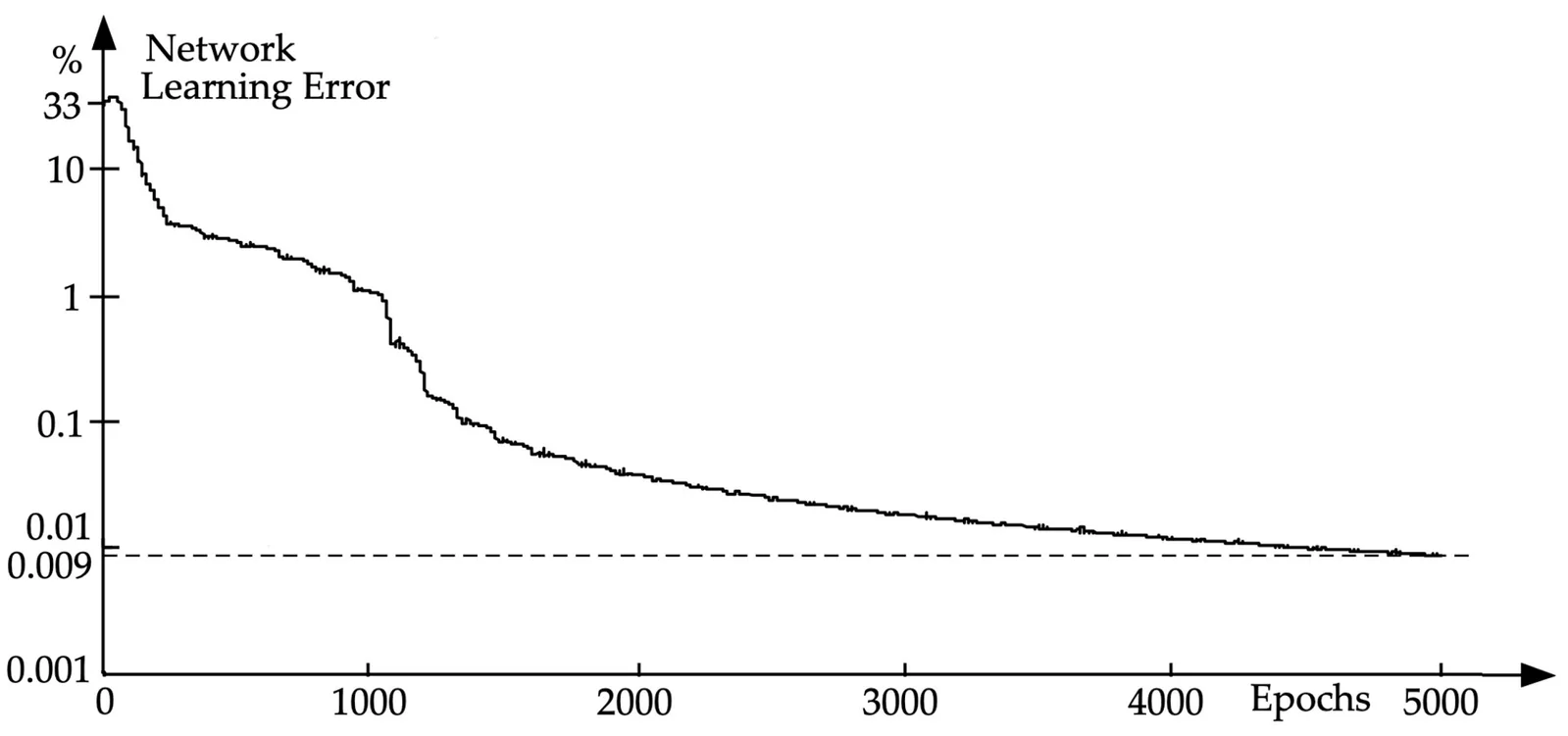
The mean square error of the learning process of a neural network assessing the risk of ship collision, marked with a dashed line.

**Figure 6:**
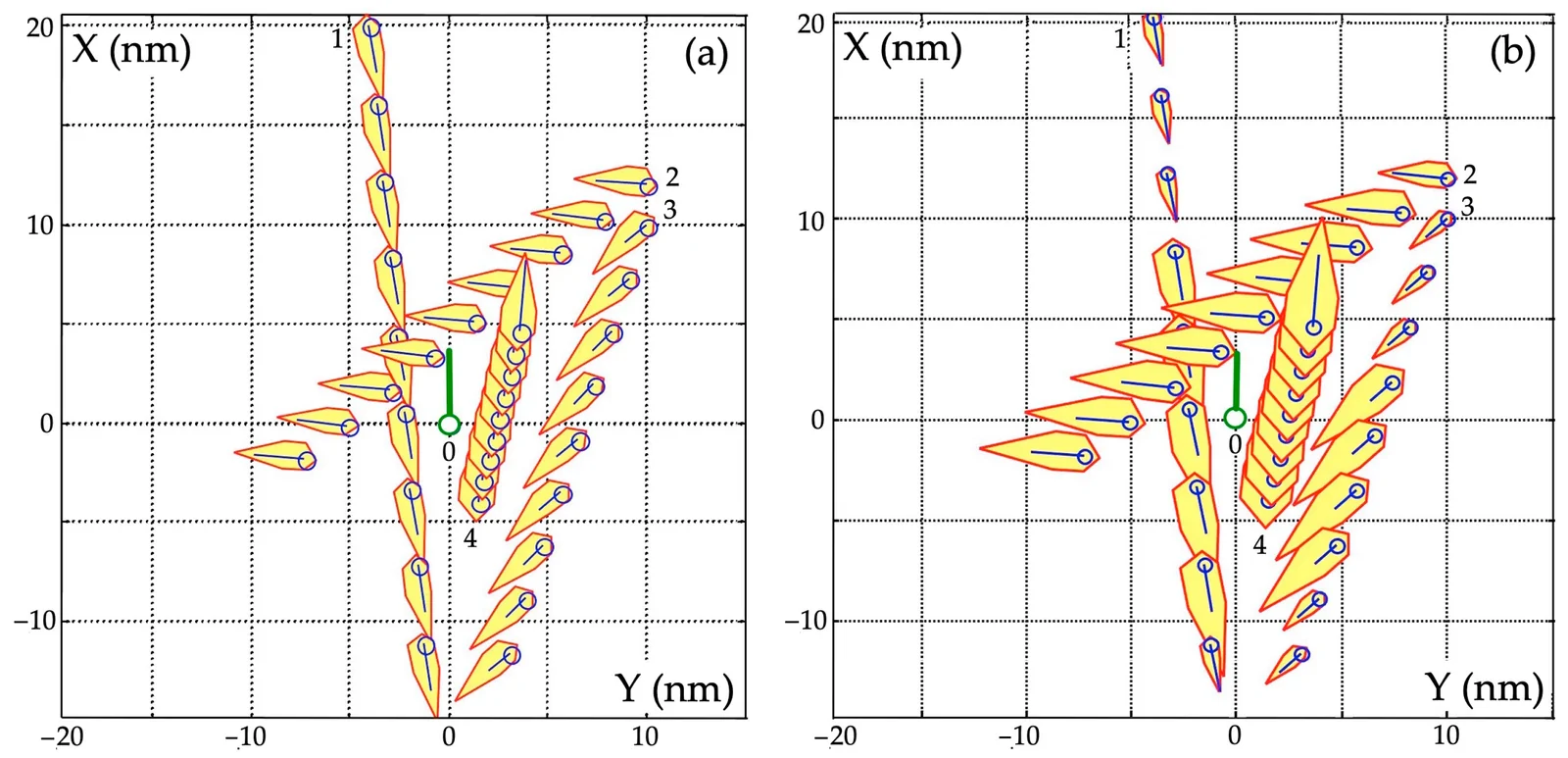
Comparison of the shape of domains generated by the inactive (**a**) and active (**b**) neural network in the situation of passing *j* = 4 ships.

**Figure 7:**
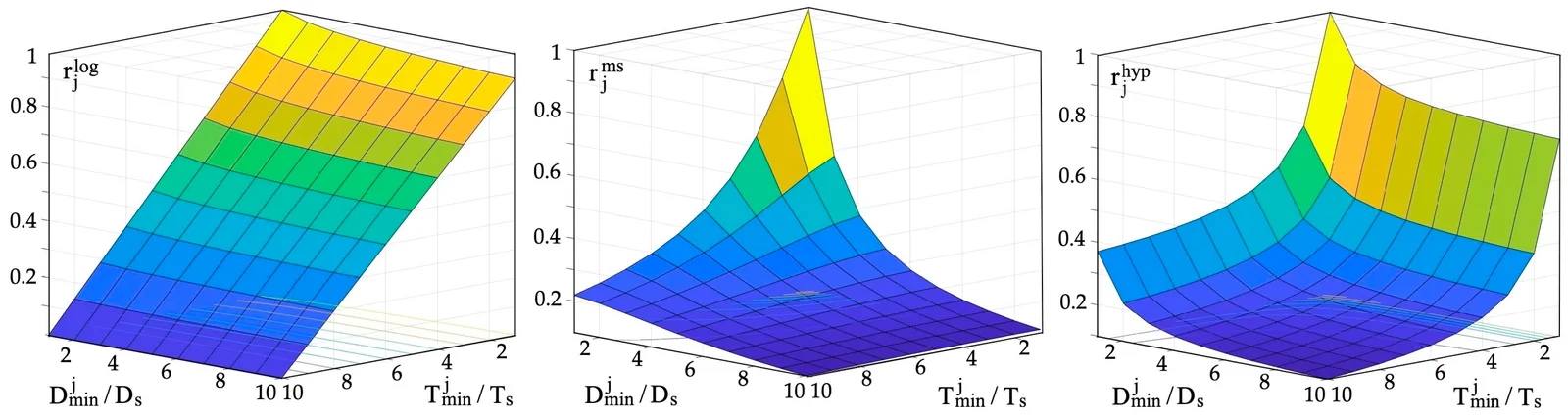
Dependence of collision risk *r_j_* on relative values of distance $D_{min}^{j}/D_{s}$ and approach time $T_{min}^{j}/T_{s}$ of the subject ship and ship *j* for the three models: logarithmic *log* (**left**); mean square *ms* (**middle**); and hyperbolic *hyp* (**right**).

**Figure 8:**
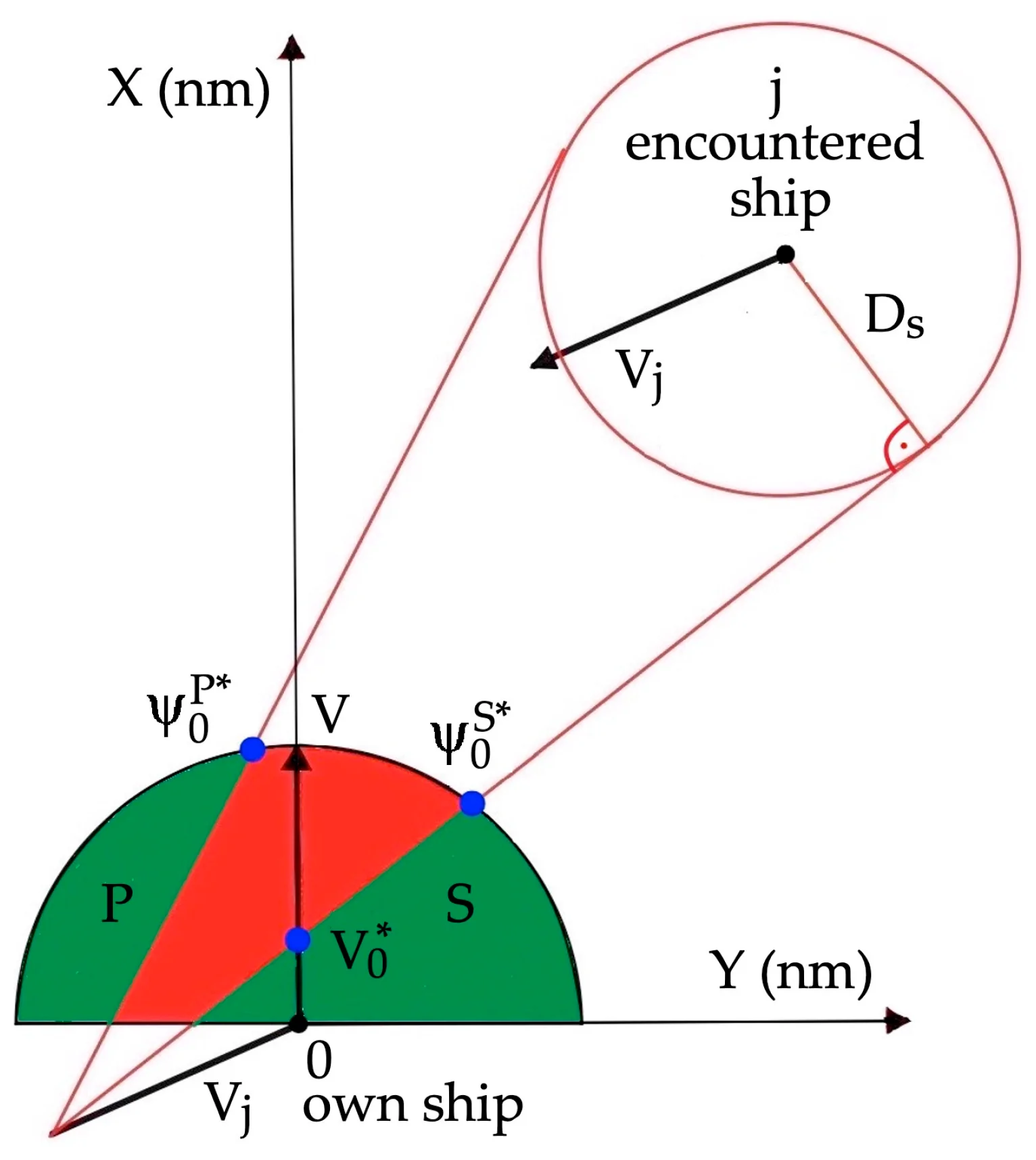
Determination of the sets of permissible maneuvers of the subject ship at 0 (green), where the safe optimal courses, ensuring the smallest deviation from the set route, to starboard and port are $\psi_{0}^{S*}$ and $\psi_{0}^{P*}$, respectively, and the optimal safe maneuver occurs at reduced speed $V_{0}^{*}$.

**Figure 9:**
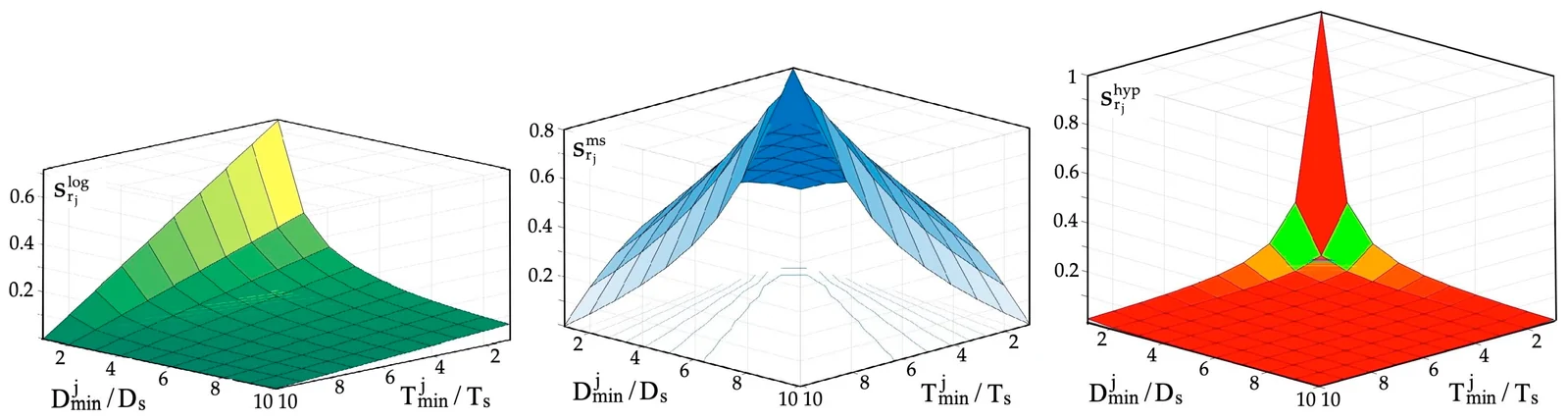
Sensitivity characteristics *s_rj_* of collision risk based on relative values of distance $D_{min}^{j}/D_{s}$ and approach time $T_{min}^{j}/T_{s}$ of the subject ship and ship *j* for the following models: logarithmic *log* (**left**); mean square *ms* (**middle**); and hyperbolic *hyp* (**right**).

**Figure 10:**
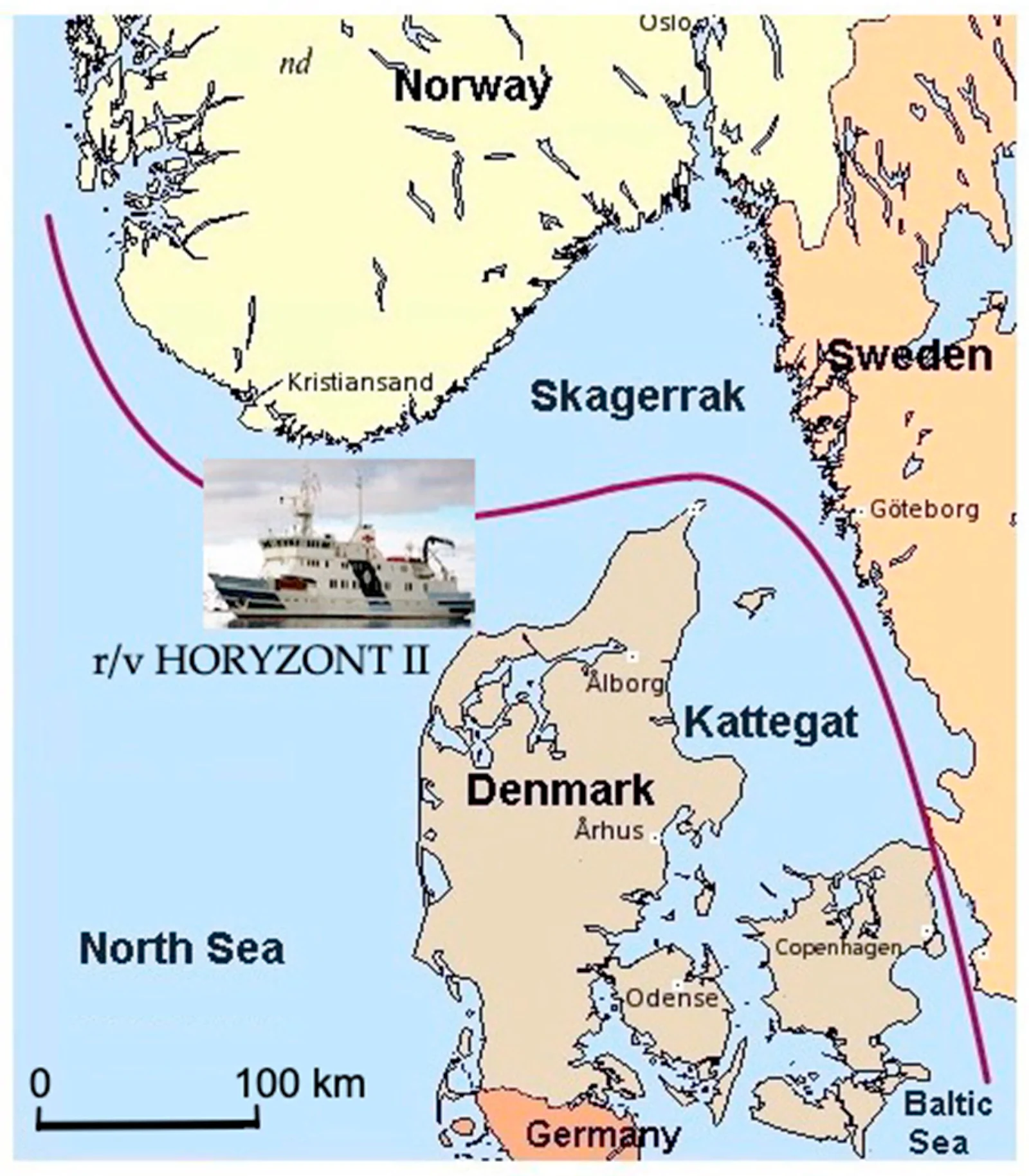
Graphical presentation of the experimental case used to record navigational situations in the Skagerrak and Kattegat Straits on the research and training ship r/v HORYZONT II.

**Figure 11:**
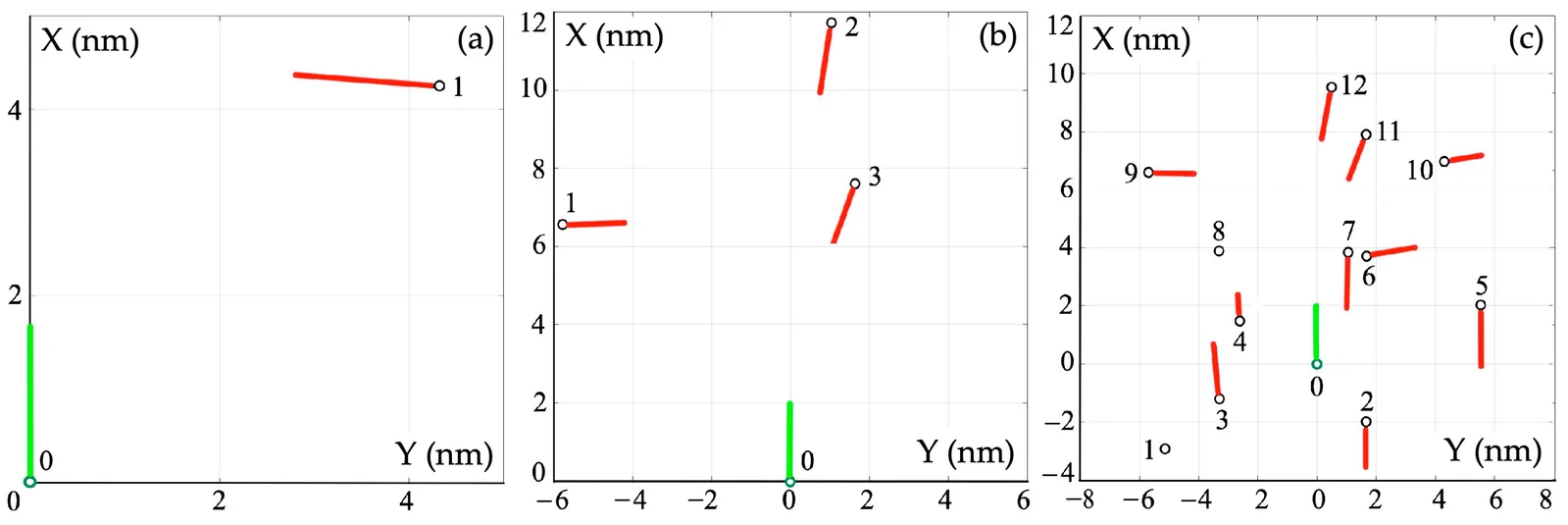
The navigational situations of the subject ship’s motion when passing *J* ships, illustrated in the form of their six-minute velocity vectors: (**a**) *J* = 1; (**b**) *J* = 3; and (**c**) *J* = 12.

**Figure 12:**
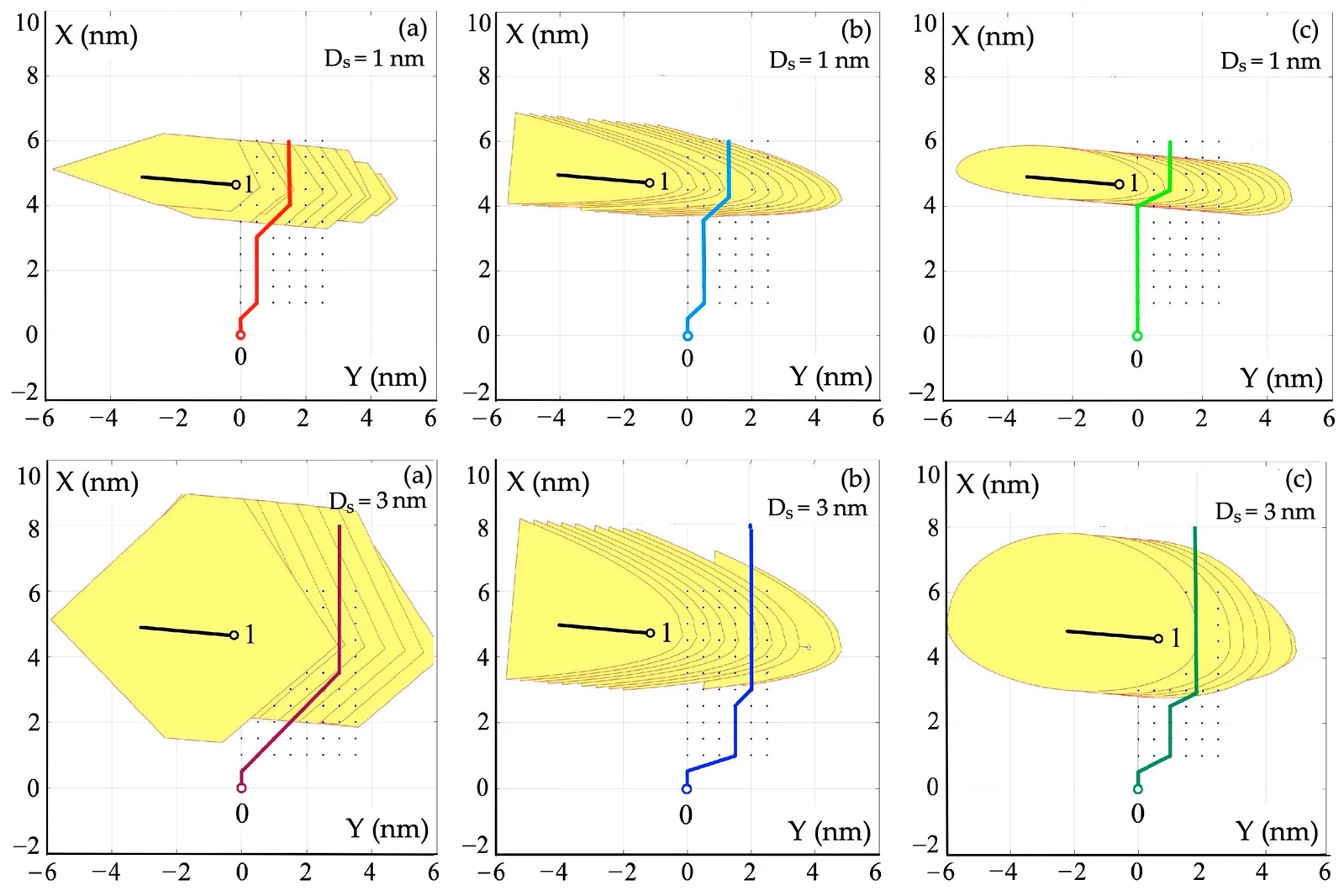
Safe trajectories of the subject ship passing one ship (*j* = 1) under conditions of good visibility at *D_s_* = 1 nm and restricted visibility at *D_s_* = 3 nm, determined via the NDC method: (**a**) NDC_h, with a hexagonal domain (red); (**b**) NDC_p, with a parabolic domain (blue); and (**c**) NDC_e, with an elliptical domain (green).

**Figure 13:**
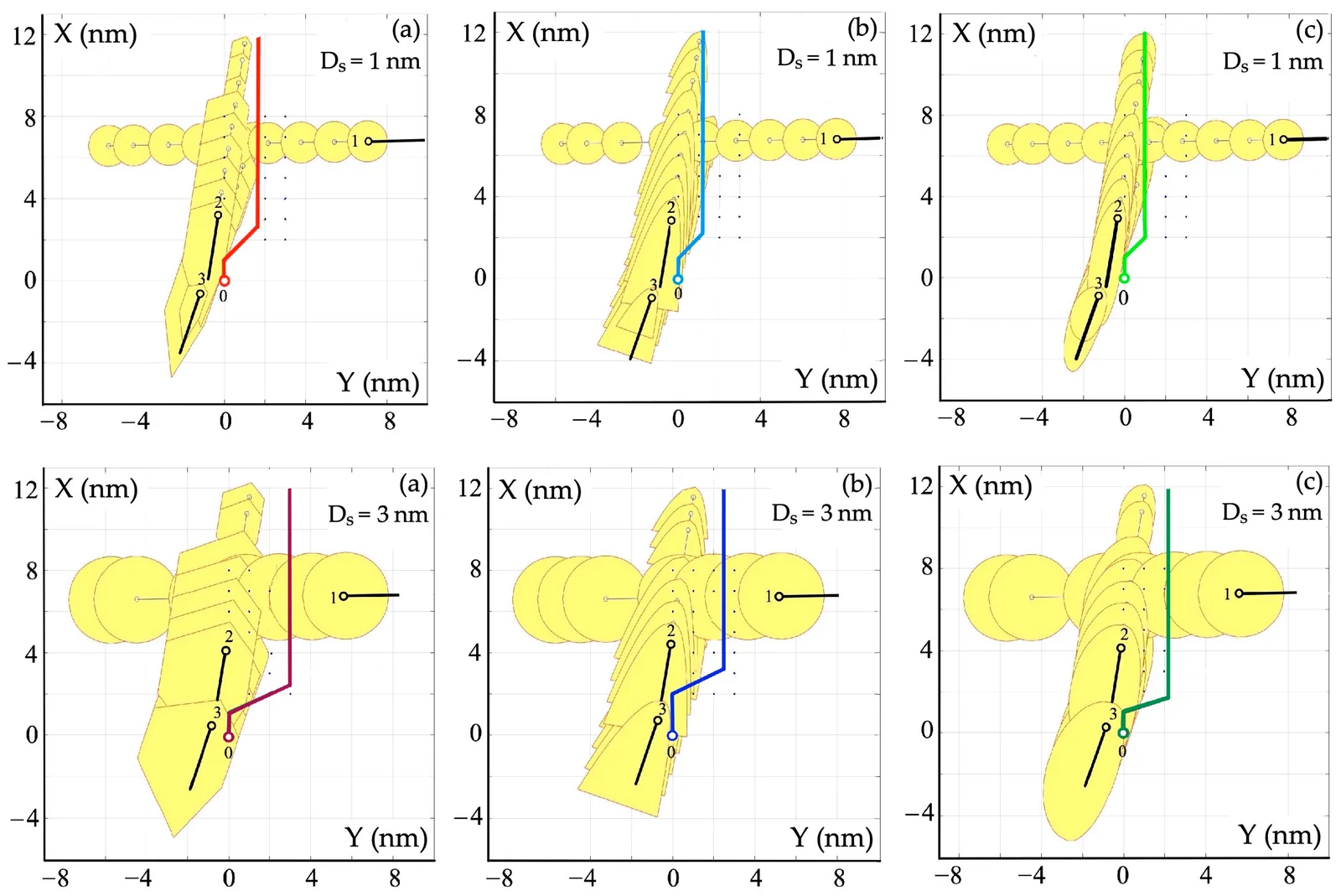
Safe trajectories of the subject ship passing three ships *(j* = 3) under conditions of good visibility at *D_s_* = 1 nm and restricted visibility at *D_s_* = 3 nm, determined via the NDC method: (**a**) NDC_h, with a hexagonal domain (red); (**b**) NDC_p, with a parabolic domain (blue); and (**c**) NDC_e, with an elliptical domain (green).

**Figure 14:**
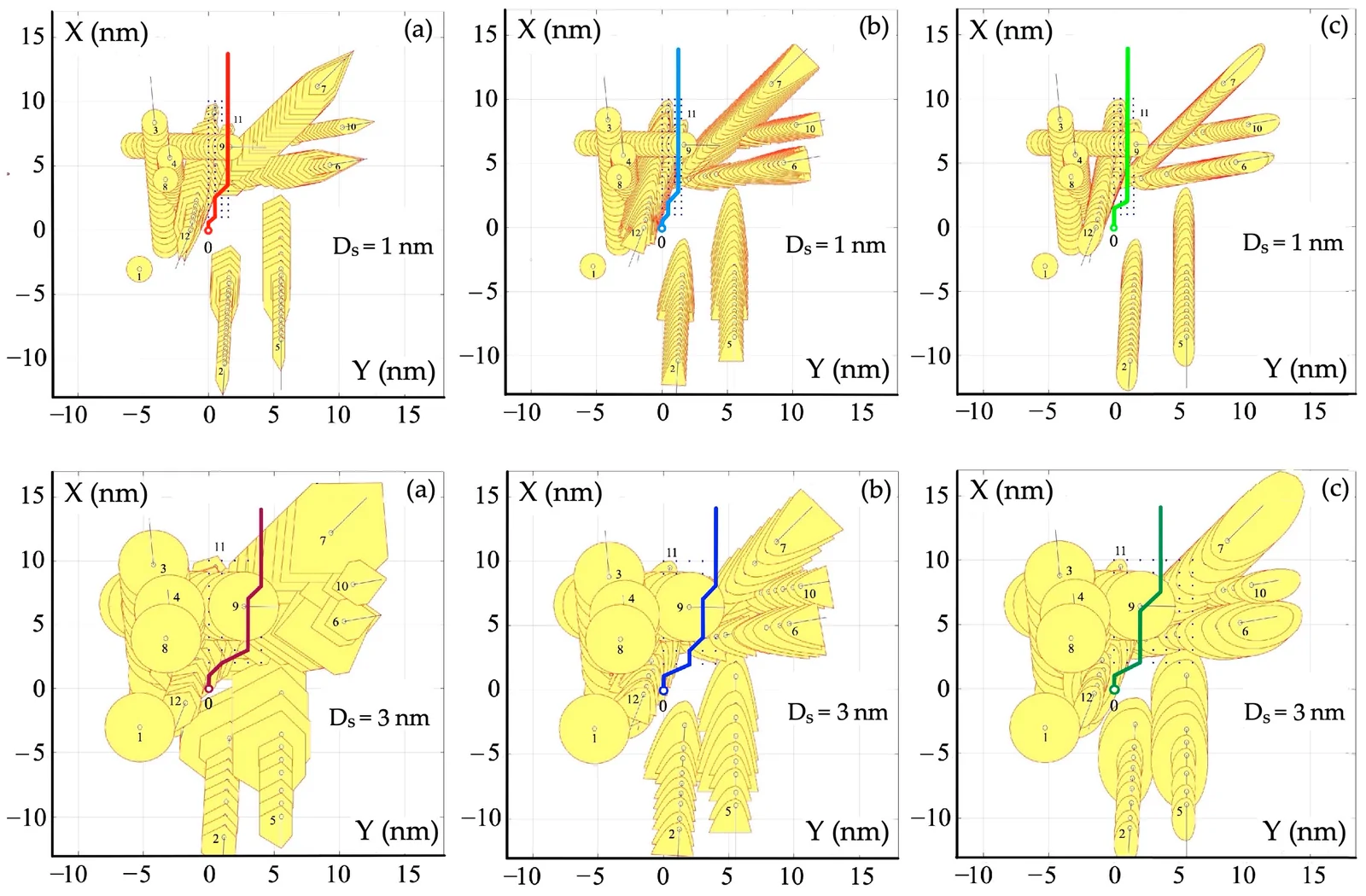
Safe trajectories of the subject ship passing twelve ships (*J* = 12) under conditions of good visibility at *D_s_* = 1 nm and restricted visibility at *D_s_* = 3 nm, determined via the NDC method: (**a**) NDC_h, with a hexagonal domain (red); (**b**) NDC_p, with a parabolic domain (blue); and (**c**) NDC_e, with an elliptical domain (green).

**Figure 15:**
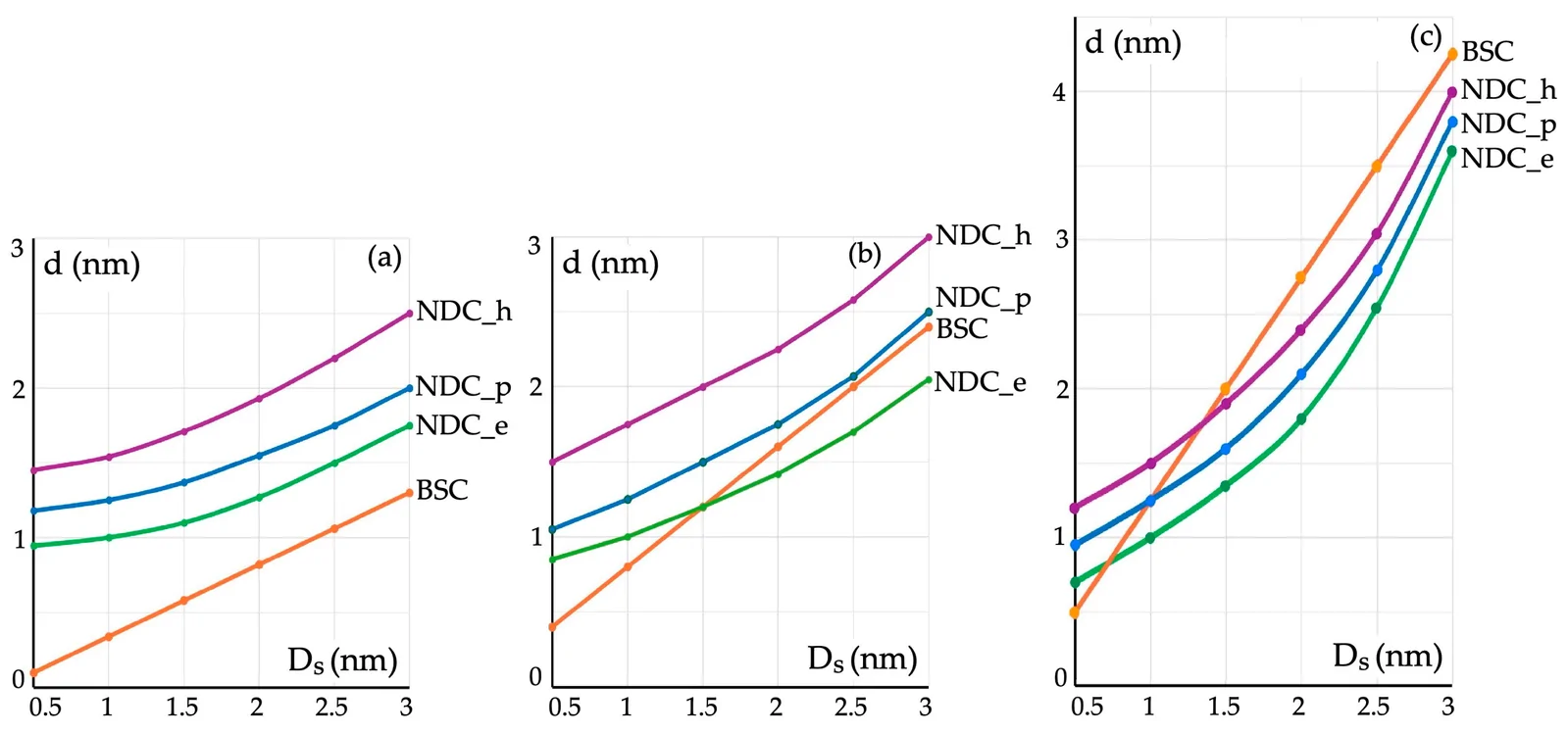
Characteristics of the final deviation *d* in the subject ship’s safe trajectory, determined according to the NDC_h, NDC_p, NDC_e, and BSC methods: (**a**) *J* = 1; (**b**) *J* = 3; and (**c**) *J* = 12.

**Figure 16:**
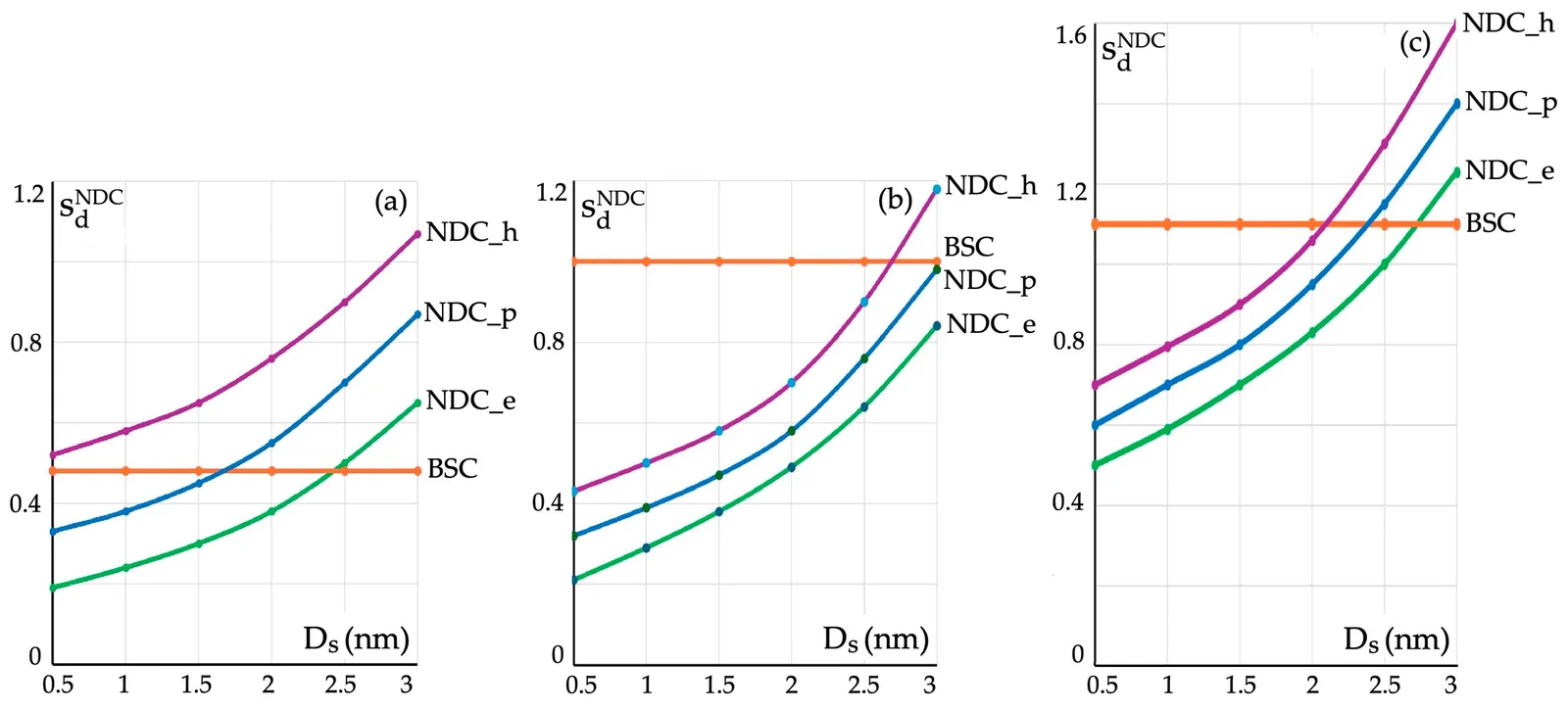
Sensitivity characteristics $s_{d}^{NDC}$ of the final deviation *d* in the subject ship’s safe trajectory to changes in the safe distance *D_s_* of the passing *J* ships: (**a**) *J* = 1; (**b**) *J* = 3; and (**c**) *J* = 12.

**Figure 17:**
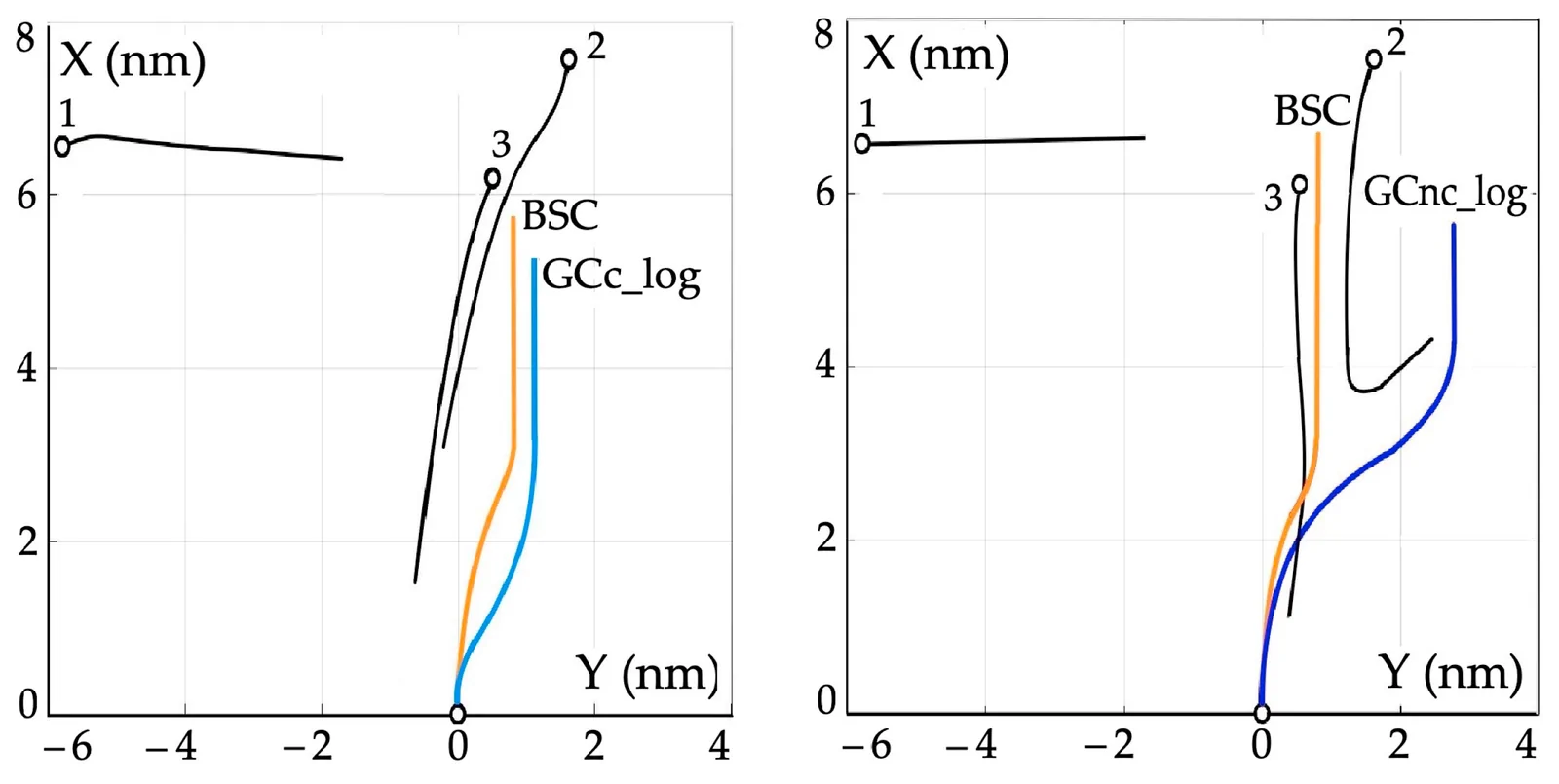
Comparison of safe ship trajectories for cooperative (**left**) and non-cooperative (**right**) collision avoidance maneuvers, determined via the GCc_log and GCnc_log methods, respectively, as well as via the BSC (basic safe control) method for comparison.

**Figure 18:**
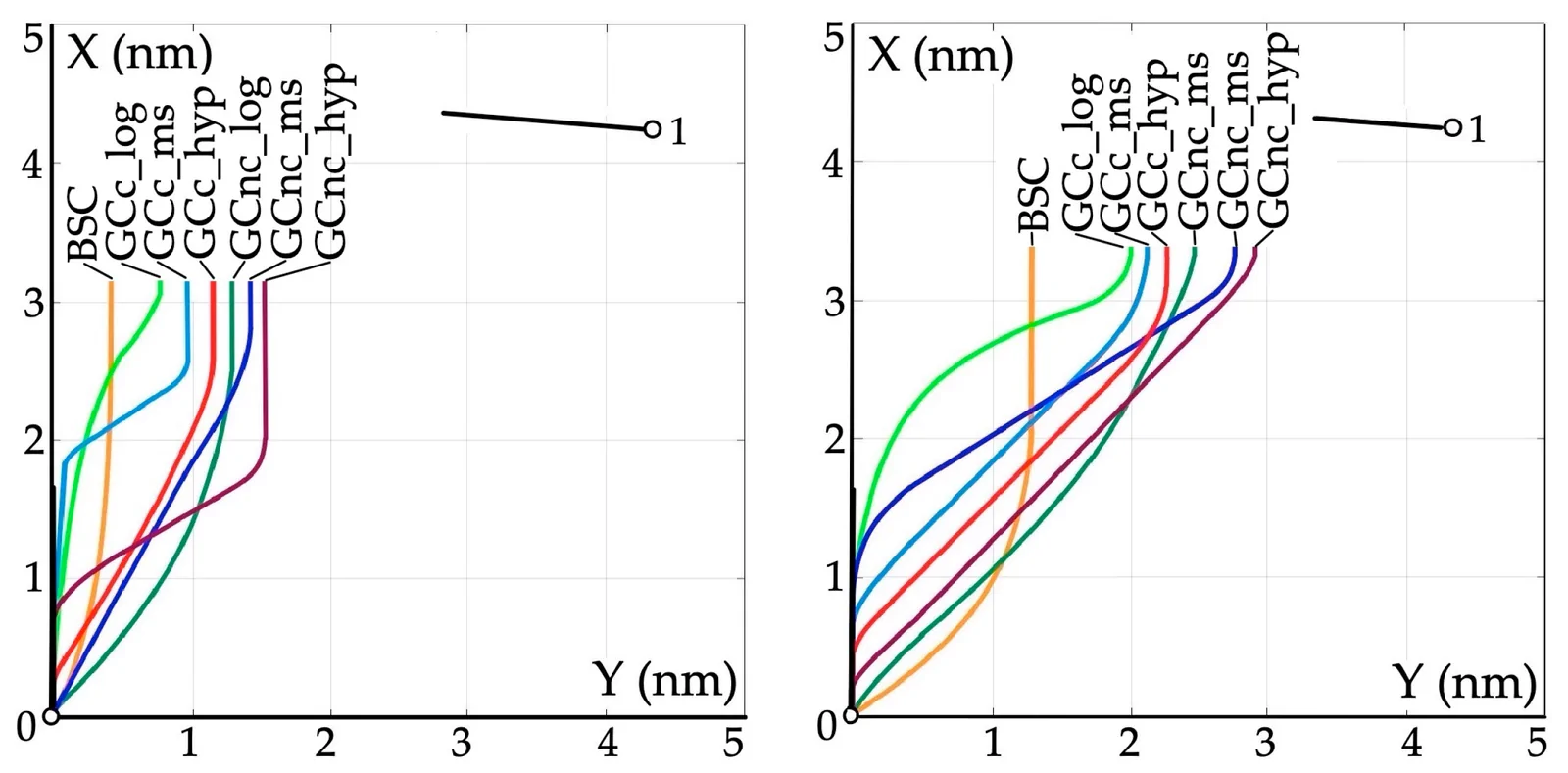
Safe trajectories of the subject ship when passing one ship (*J* = 1) under conditions of good visibility at *D_s_* = 1 nm (**left**) and restricted visibility at *D_s_* = 3 nm (**right**), calculated using the cooperative and non-cooperative game control methods with the collision risk model *r_j_*—logarithmic GCc_log and GCnc_log, mean square GCc_ms and GCnc_ms, and hyperbolic GCc_hyp and GCnc_hyp; for comparison, the trajectories of the basic safe control method (*BSC*) are presented.

**Figure 19:**
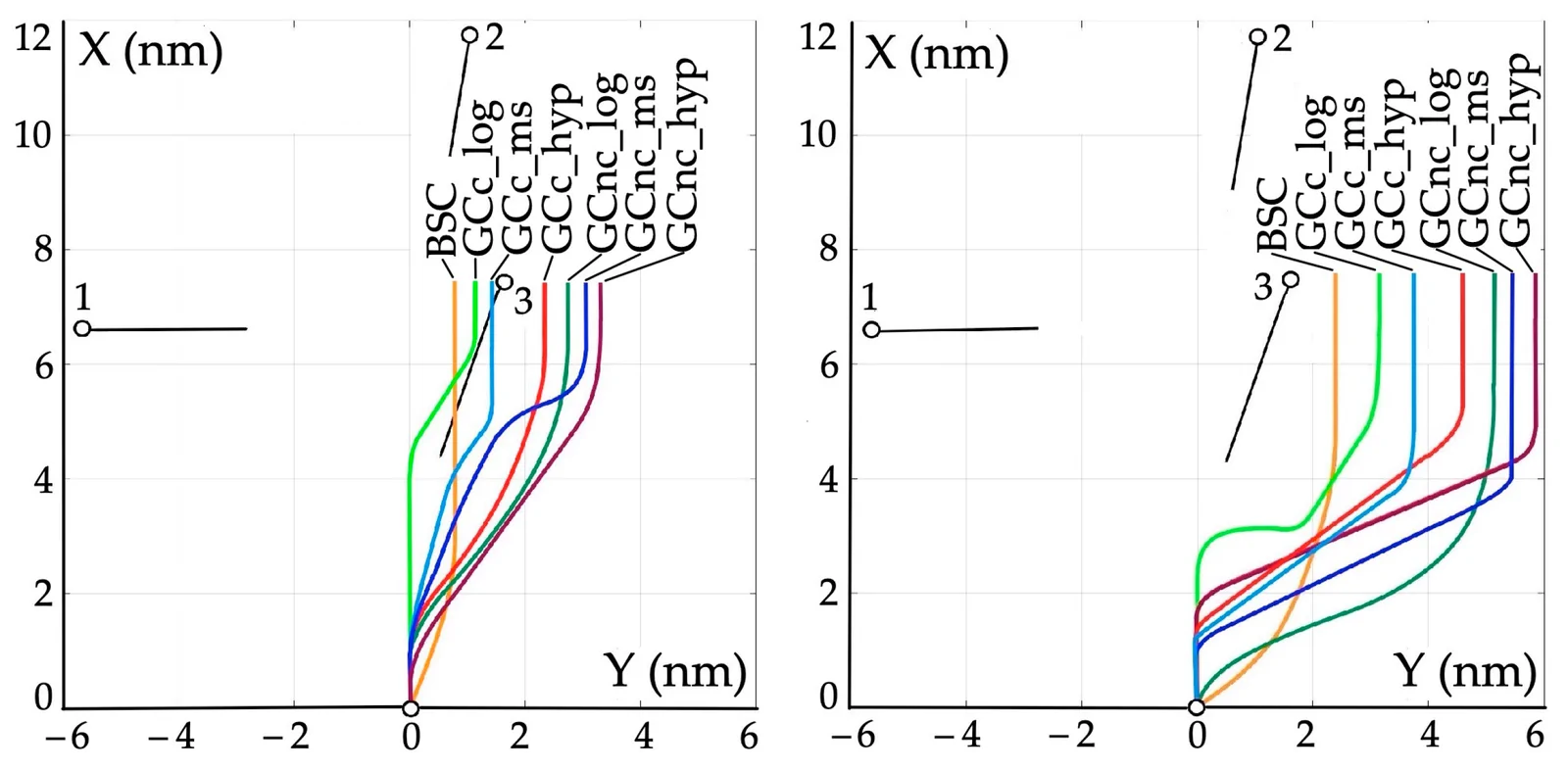
Safe trajectories of the subject ship when passing three ships (*J* = 3) under conditions of good visibility at *D_s_* = 1 nm (**left**) and restricted visibility at *D_s_* = 3 nm (**right**), calculated using the cooperative and non-cooperative game control methods with the collision risk model *r_j_*—logarithmic GCc_log and GCnc_log, mean square GCc_ms and GCnc_ms, and hyperbolic GCc_hyp and GCnc_hyp; for comparison, the trajectories of the basic safe control method (BSC) are presented.

**Figure 20:**
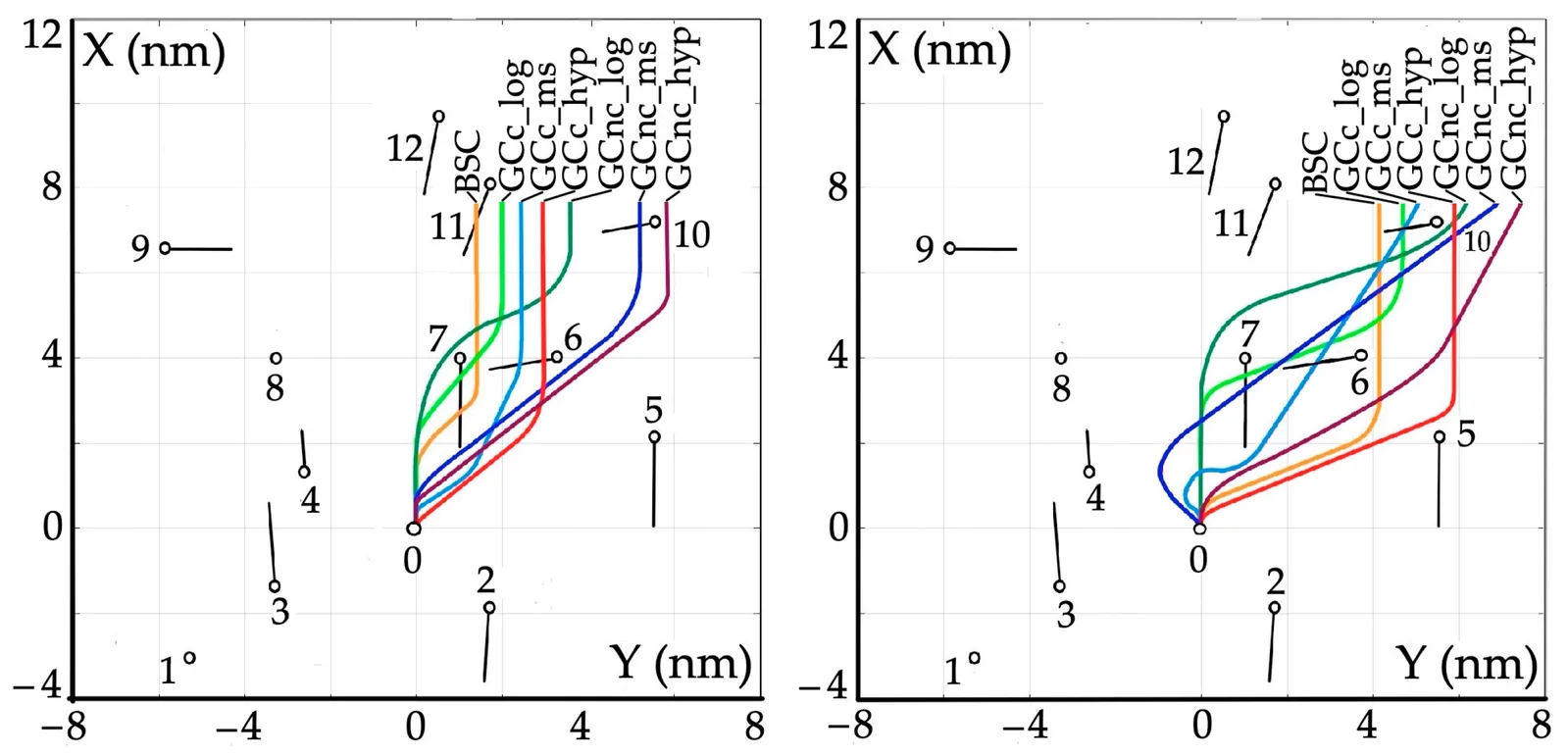
Safe trajectories of the subject ship when passing twelve ships (*J* = 12) under conditions of good visibility at *D_s_* = 1 nm (**left**) and restricted visibility at *D_s_* = 3 nm (**right**), calculated using the cooperative and non-cooperative game control methods with the collision risk model *r_j_*—logarithmic GCc_log and GCnc_log, mean square GCc_ms and GCnc_ms, and hyperbolic GCc_hyp and GCnc_hyp; for comparison, the trajectories of the basic safe control method (BSC) are presented.

**Figure 21:**
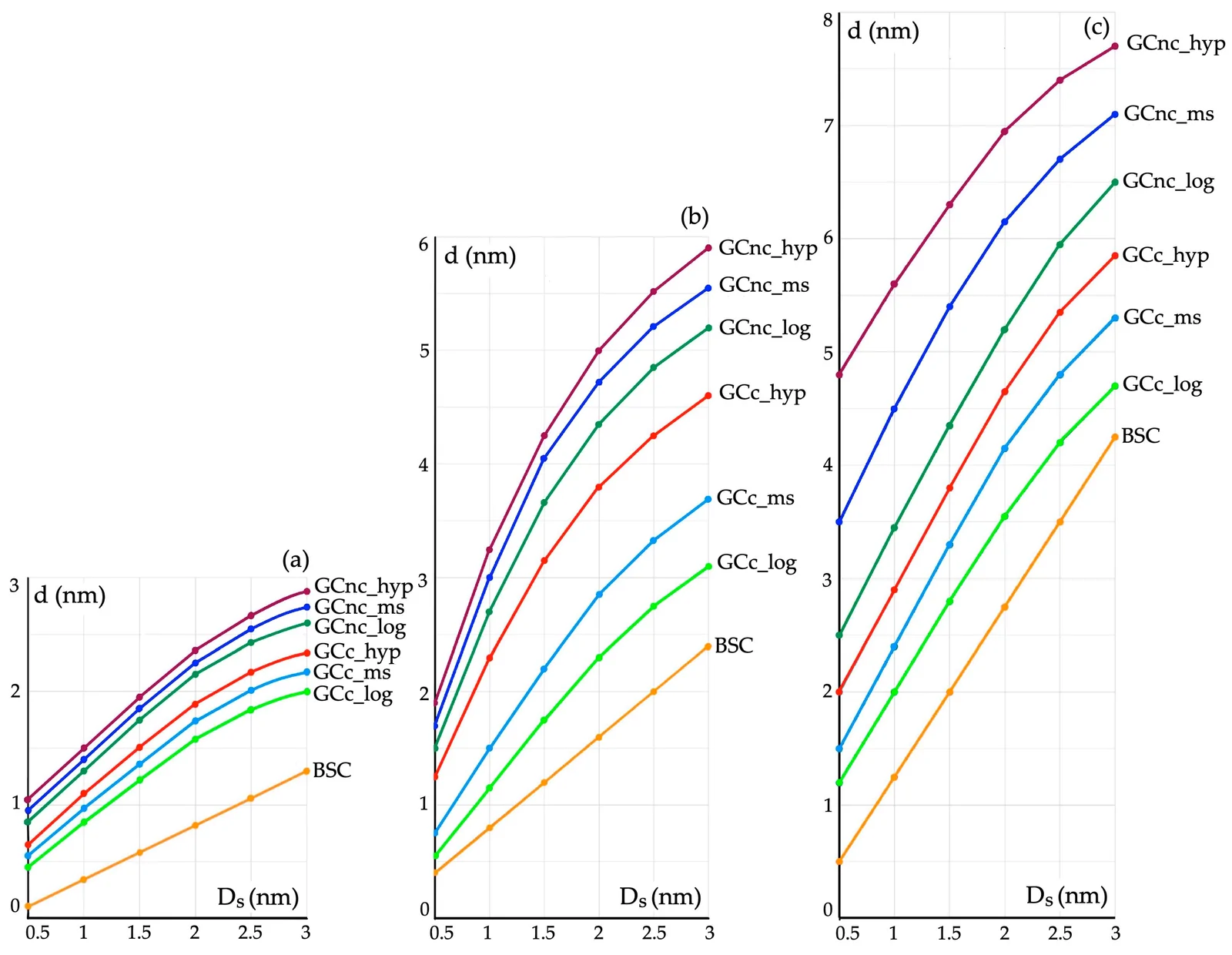
Characteristics of the final deviation *d* of the subject ship’s safe trajectory as a function of the safe passing distance *D_s_* for the cooperative (GCc) and non-cooperative (GCnc) control methods, using the logarithmic *log*, mean square *ms*, and hyperbolic *hyp* collision risk model: (**a**) *J* = 1; (**b**) *J* = 3; and (**c**) *J* = 12.

**Figure 22:**
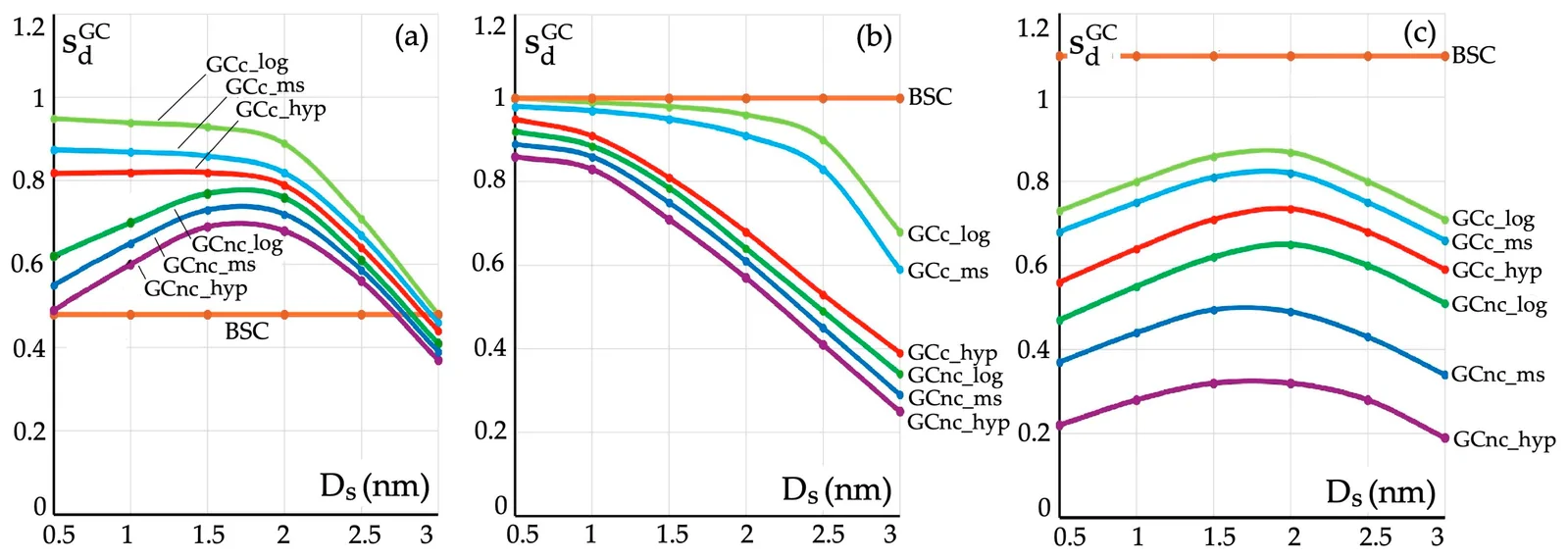
Sensitivity characteristics $s_{d}^{GC}$ of the final deviation *d* of the subject ship’s safe trajectory to changes in the safe distance *D_s_* when passing *J* ships: (**a**) *J* = 1; (**b**) *J* = 3; and (**c**) *J* = 12.

**Figure 23:**
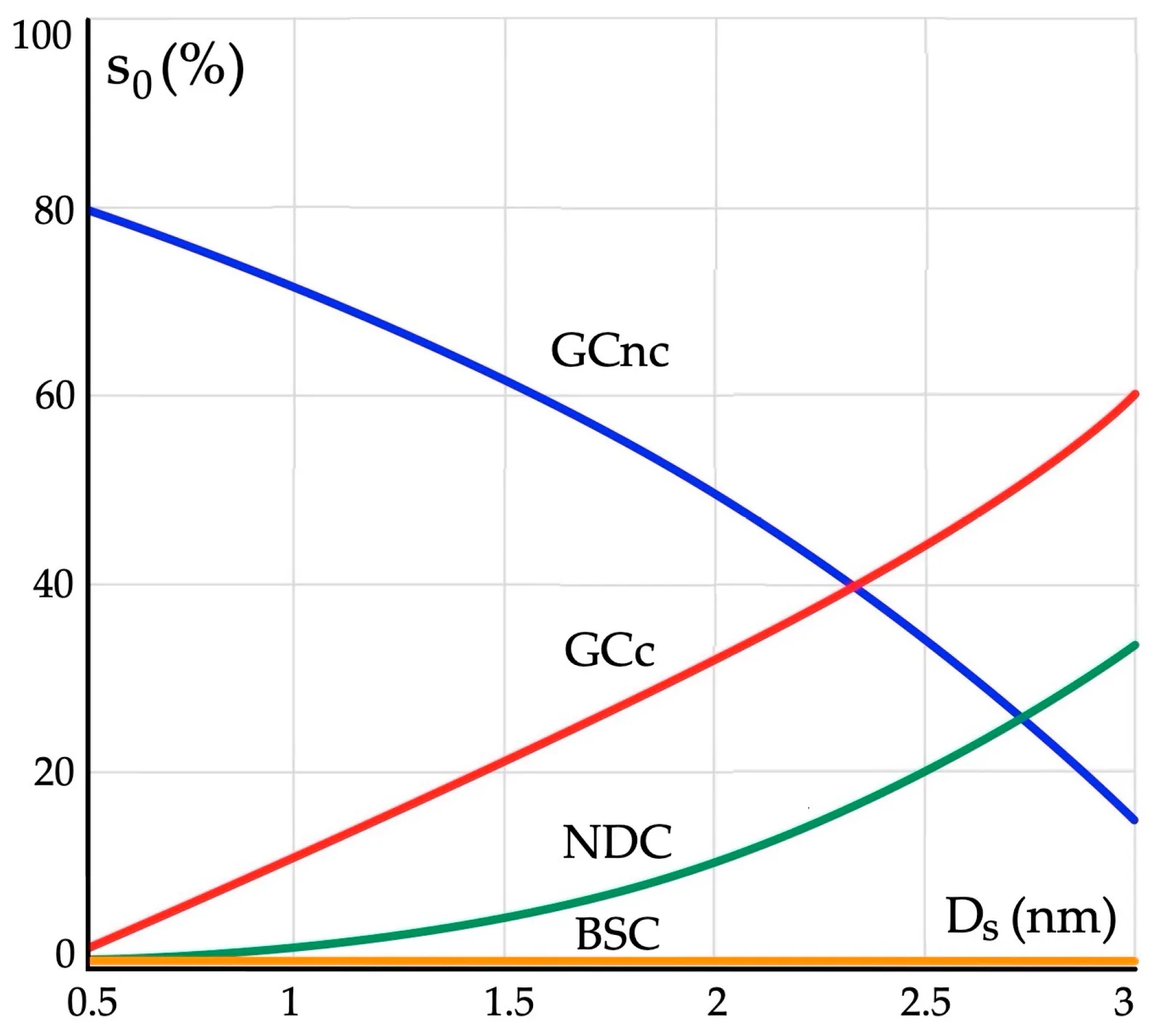
Effectiveness characteristics *s*_0_ of the algorithms for the safe control of the subject ship, taking into account the movement of three other ships, depending on the state of the navigation situation represented by the value of the safe passing distance *D_s_* according to COLREG, for the methods of GCnc, GCc, NDC, and, as a reference, basic safe control (BSC).

**Table 1:** Data describing the navigational situations of the subject ship’s movement and the *J* ships it passes.

Situation	Object Number *j*	Speed *V_j_* (kn)	Course y*_j_* (deg)	Coordinate *X_j_* (nm)	Coordinate *Y_j_* (nm)	DCPA (nm)	TCPA (min)
*J* = 1	0	16.0	0	0	0	-	-
	1	14.0	275	4.2	4.3	0.1	17
*J* = 3	0	20.0	0	0	0	-	-
	1	14.5	89	6.4	−5.6	0.6	22
	2	16.2	189	11.2	1.0	0.5	13
	3	16.1	199	7.27	1.5	0.1	18
*J* = 12	0	20.0	0	0	0	-	-
	1	0	180	−3.1	−5.2	5.8	0
	2	15.9	183	−1.9	1.7	2.5	0
	3	18.1	355	−1.2	−3.2	3.2	0
	4	7.8	355	1.5	−2.6	2.5	9
	5	19.9	180	2.0	5.6	5.5	0
	6	14.5	80	3.6	1.7	2.5	5
	7	19.5	181	3.8	0.9	1.1	6
	8	0	1	3.9	−3.2	3.3	12
	9	13.9	91	6.4	−5.7	0.4	18
	10	11.3	80	6.9	4.3	7.1	6
	11	16.1	201	7.8	1.7	0.5	13
	12	17.1	191	9.3	0.5	0.7	15

**Table 2:** Comparison of performance results for the neural-dynamic control, NDC, with basic safe control, BSC.

Number of Ships *J*	Safe Distance *D_s_* (nm)	Control Method	Domain	Final Deviation *d* (nm)	Computation Time t*_c_* (s)
1	1.0	Neural-dynamic control	Hexagonal	1.44	16
			Parabolic	1.19	15
			Elliptical	0.97	15
		Non-game BSC	-	0.11	2
	3.0	Neural-dynamic control	Hexagonal	2.52	15
			Parabolic	2.02	14
			Elliptical	1.80	14
		Non-game BSC	-	1.30	2
3	1.0	Neural-dynamic control	Hexagonal	1.48	14
			Parabolic	1.08	15
			Elliptical	0.86	15
		Non-game BSC	-	0.40	6
	3.0	Neural-dynamic control	Hexagonal	3.02	12
			Parabolic	2.52	13
			Elliptical	2.05	13
		Non-game BSC	-	2.41	6
12	1.0	Neural-dynamic control	Hexagonal	1.19	6
			Parabolic	0.94	7
			Elliptical	0.68	7
		Non-game BSC	-	0.50	18
	3.0	Neural-dynamic control	Hexagonal	4.00	5
			Parabolic	3.78	6
			Elliptical	3.56	6
		Non-game BSC	-	4.21	18

**Table 3:** Comparison of performance results for the cooperative and non-cooperative game control algorithms—GC, with non-game basic safe control—BSC.

Number of Ships *J*	Safe Distance *D_s_* (nm)	Control Method	Collision Risk Model	Final Deviation *d* (nm)	Computation Time t*_c_* (s)
1	1.0	Cooperative GC	Logarithmic	0.44	12
			Mean Square	0.56	12
			Hyperbolic	0.64	12
		Non-cooperative GC	Logarithmic	0.84	15
			Mean Square	0.96	15
			Hyperbolic	1.04	15
		Non-game BSC	-	0.12	2
	3.0	Cooperative GC	Logarithmic	1.96	14
			Mean Square	2.16	14
			Hyperbolic	2.32	14
		Non-cooperative GC	Logarithmic	2.36	17
			Mean Square	268	17
			Hyperbolic	2.88	17
		Non-game BSC	-	1.28	2
3	1.0	Cooperative GC	Logarithmic	0.56	19
			Mean Square	0.74	19
			Hyperbolic	1.20	19
		Non-cooperative GC	Logarithmic	1.44	21
			Mean Square	1.68	21
			Hyperbolic	1.88	21
		Non-game BSC	-	0.42	6
	3.0	Cooperative GC	Logarithmic	3.08	24
			Mean Square	3.64	24
			Hyperbolic	4.52	24
		Non-cooperative GC	Logarithmic	5.16	27
			Mean Square	5.52	27
			Hyperbolic	5.84	27
		Non-game BSC	-	2.36	6
12	1.0	Cooperative GC	Logarithmic	1.20	58
			Mean Square	1.48	58
			Hyperbolic	2.00	58
		Non-cooperative GC	Logarithmic	2.40	61
			Mean Square	3.49	61
			Hyperbolic	4.78	61
		Non-game BSC	-	0.48	18
	3.0	Cooperative GC	Logarithmic	4.64	72
			Mean Square	5.27	72
			Hyperbolic	5.80	72
		Non-cooperative GC	Logarithmic	6.50	81
			Mean Square	7.08	81
			Hyperbolic	7.76	81
		Non-game BSC	-	4.16	18

## Data Availability

All data supporting the findings of this study are available within the article.
